# Organic Optoelectronic Synapses for Sound Perception

**DOI:** 10.1007/s40820-023-01116-3

**Published:** 2023-05-24

**Authors:** Yanan Wei, Youxing Liu, Qijie Lin, Tianhua Liu, Song Wang, Hao Chen, Congqi Li, Xiaobin Gu, Xin Zhang, Hui Huang

**Affiliations:** 1 College of Materials Science and Opto-Electronic Technology and Center of Materials Science and Optoelectronics Engineering, CAS Center for Excellence in Topological Quantum Computation, CAS Key Laboratory of Vacuum Physic, University of Chinese Academy of Sciences, Beijing, 100049 People’s Republic of China; 2 School of Materials Science and Engineering, Peking University, Beijing, 100871 People’s Republic of China

**Keywords:** Organic optoelectronic synapse, Sound perception, Recognition factor, Impedance spectroscopy, Interfacial layer

## Abstract

The neuromorphic systems for sound perception is under highly demanding for the future bioinspired electronics and humanoid robots. However, the sound perception based on volume, tone and timbre remains unknown. Herein, organic optoelectronic synapses (OOSs) are constructed for unprecedented sound recognition. The volume, tone and timbre of sound can be regulated appropriately by the input signal of voltages, frequencies and light intensities of OOSs, according to the amplitude, frequency, and waveform of the sound. The quantitative relation between recognition factor (*ζ*) and postsynaptic current (*I* = *I*_light_ − *I*_dark_) is established to achieve sound perception. Interestingly, the bell sound for University of Chinese Academy of Sciences is recognized with an accuracy of 99.8%. The mechanism studies reveal that the impedance of the interfacial layers play a critical role in the synaptic performances. This contribution presents unprecedented artificial synapses for sound perception at hardware levels.

**Supplementary Information:**

The online version contains supplementary material available at 10.1007/s40820-023-01116-3.

## Highlights

- The organic optoelectronic synapse achieves unprecedented sound perception on volume, tone and timbre simultaneously.
- The quantitative relationship between the interfacial layers and synaptic performances is clarified.

**Supplementary Information:**

The online version contains supplementary material available at 10.1007/s40820-023-01116-3.

## Graphical abstract

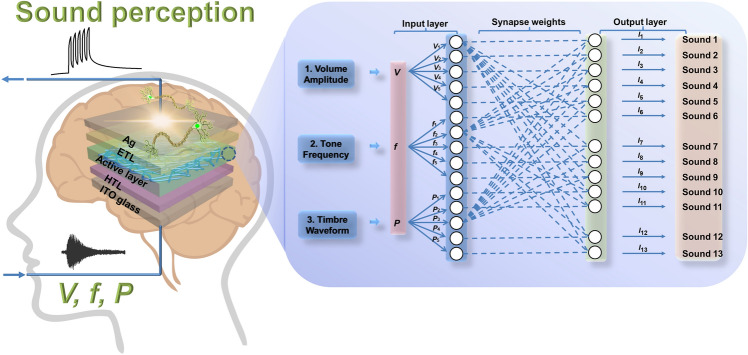

## Introduction

Human beings interact with their environment by perceiving and responding to external stimuli such as light, sound, pressure, and chemicals [1–4]. Among the external stimuli, sounds can deliver rich information due to the acoustical features of voice including volume, tone, and timbre [5–10]. First, the volume referred to the size of a sound can be measured in decibels (dB), which is used for audiometry test and determining hearing thresholds [11]. Moreover, the tone is related to the frequencies of the sounds that are divided into infrasound waves (< 20 Hz), audible sound waves (20–20 kHz), and ultrasonic waves (> 20 kHz). At last, the timbre related to the waveform represents the uniqueness of sound. According to the timbre, one can evaluate the physical characteristics of different protagonists, including genders, approximate ages and sizes, and associate the identities to the different voices [5, 6]. Briefly, accurate perception of sound with volume, tone and timbre is instrumental for hearing protection, natural disasters prediction, and medical applications.

In the era of artificial intelligence, synapses for learning, calculating, and memorizing information are rising for the future bioinspired electronic devices and humanoid robots [1, 12–18]. Many efforts have been made to construct synaptic devices for sound perception. For example, artificial van der Waals hybrid synapse based on a hardware neural-network was used in acoustic pattern to recognize the word of voice by changing the input voltage [19]. Furthermore, versatile electrical synapses was used for artificial auditory sensory systems by modulating synaptic decay of single organic synaptic transistor [20]. Also, artificial auditory pathway for intelligent neuromorphic computing and sound detection was realized via synaptic transistors [21]. However, the artificial intelligence for recognizing and memorizing the volume, tone and timbre of sound simultaneously has never been achieved in hardware level.

Organic optoelectronic synapses (OOSs) were one of the important technologies to simulate artificial intelligence due to their advantages of easily-tunable optical-response range, solution processability, mechanic flexibility, etc. Various mechanisms have been implemented to achieve synaptic characteristics for OOSs, including charge trapping, conductive filament, ion migration, floating gate, and dipole alignment [3]. Among them, the charge trapping at interfaces can induce change in the conductivity, affording in postsynaptic responses [3]. For instance, the charge trapping at the chlorophyll/organic semiconductors and the dielectric/organic semiconductors interfaces caused the multifunction of photodetectors and light stimulated synaptic transistors [22]. Also, the accumulation of trapped carriers in the active layer/dielectric layer causes the change of local surface potential with light intensities, leading to active photoadaptation behaviour in a single device [14]. However, the relationship between the interfacial layers and synaptic performances are still elusive.

**Fig. 1:**
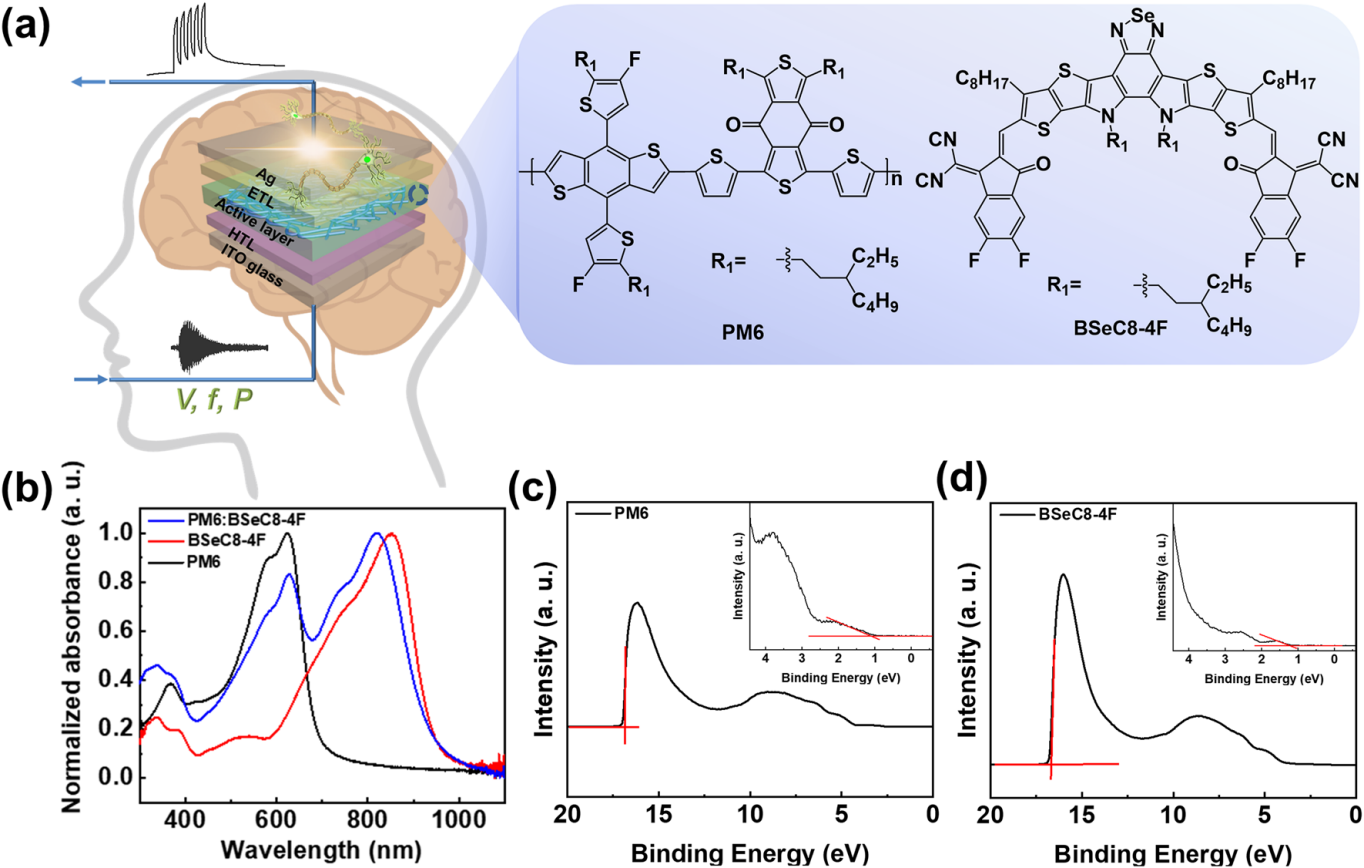
**a** Device structure of ITO/PEDOT:PSS/D:A/PDINN/Ag (Device I) and chemical structures of the donor and acceptor materials in device. **b** Optical absorption spectra of PM6, BSeC8-4F, and PM6:BSeC8-4F films. **c**–**d** UPS measurements of PM6 and BSeC8-4F films

Here, OOSs with a device structure of ITO/PEDOT:PSS/D:A/PDINN/Ag (Fig. 1a) were constructed for accurate sound perception. The volume, tone and timbre of sound were regulated systematically by tuning the input signal of voltages, frequencies and light intensities of OOSs, according to the amplitude, frequency, and waveform of the sound. Impressively, a novel parameter of recognition factor (*ζ*) was proposed to establish quantitative relationship with postsynaptic current (*I* = *I*_light _− *I*_dark_), which exhibited high accuracy for sound perception. The mechanism studies revealed the key role of the impedance of the interfacial layers, which is correlated with the synaptic performances.

## Experimental Section

### Materials

All reagents were purchased from Inno-chem, *J&K*, 3A Chemicals, Derthon, Energy Chemical Co., Ltd., Acros, and Hyper Inc., unless specified and used as received. PM6, P3HT, PC_71_BM, and ITIC were provided by Solarmer Materials Inc Beijing, while PBDB-T was provided by Ltd. Shanghai Zhuxing Optoelectronic Technology Co., Ltd.

### Device Fabrication

Device I (ITO/PEDOT:PSS/D:A/PDINN/Ag): After treated by UV-Ozone for 20 min, PEDOT:PSS (Clevios P VP Al 4083) was spin-coated onto clean ITO-coated glass substrates at 3200 rpm and baked at 150 °C for 15 min. A CHCl_3_ solution of donor (7 mg mL^−1^ for PM6) and acceptor was spin-coated onto the PEDOT:PSS layer. Afterwards, the film was annealed at 100 °C for 8 min. Then, a thin PDINN layer was spin-coated on top of the active layer at 3000 rpm, followed by the deposition of Ag (100 nm) (evaporated through a shadow mask).

Device II (ITO/PEDOT:PSS/D:A/Ag): After treated by UV-Ozone for 20 min, PEDOT:PSS (Clevios P VP Al 4083) was spin-coated onto clean ITO-coated glass substrates at 3200 rpm and baked at 150 °C for 15 min. A CHCl_3_ solution of donor (7 mg mL^−1^ for PM6) and acceptor was spin-coated onto the PEDOT:PSS layer, followed by the deposition of Ag (100 nm) (evaporated through a shadow mask).

Device III (ITO/D:A/PDINN/Ag): After treated by UV-Ozone for 20 min, a CHCl_3_ solution of donor (7 mg mL^−1^ for PM6) and acceptor was spin-coated onto the ITO-coated glass substrates. A thin PDINN layer was spin-coated on top of the active layer, followed by the deposition of Ag (100 nm) (evaporated through a shadow mask).

Device IV (ITO/D:A/Ag): After treated by UV-Ozone for 20 min, a CHCl_3_ solution of donor (7 mg mL^−1^ for PM6) and acceptor was spin-coated onto the ITO-coated glass substrates, followed by the deposition of Ag (100 nm) (evaporated through a shadow mask).

### Characterizations

The synaptic signals of organic optoelectronic synaptic devices were measured by KEYSIGHT B1500A. The current density–voltage (*J*–*V*) curves were measured with a computer-controlled Keithley 2450 Source Measure Unit under AM 1.5G white light source, the optical power at the sample was 100 mW cm^−2^ (Enlitech). UV–visible absorption spectra was carried out with a Cary 60 spectrometer, and all film samples were spin-casting on quartz glass substrates. GIWAXS measurements were conducted at XEUSS SAXS/WAXS equipment. Samples were prepared on Si substrates using same solutions as those used in devices. Atomic force microscopy (NTEGRA Solaris) was used to characterize the morphology of device in the tapping mode. The ultraviolet photoelectron spectroscopy (UPS, AXIS SUPRA, Kratos) was performed under a He I source with a 21.22 eV photoemission energy. The impedance-voltage (*Z*–*V*) profile was tested by electrochemical workstation (chi760e). ^1^H and ^13^C NMR spectra were recorded on a JEOL JNM-ECZ500R (500 MHz) spectrometer.

## Results and Discussion

A new electron acceptor 2,2′-((2Z,2′Z)-((12,13-bis(3-ethylheptyl)-3,9-dioctyl-12,13-dihydro-[1,2,5]selenadiazolo[3,4-e]thieno[2″,3″:4′,5′]thieno[2′,3′:4,5]pyrrolo[3,2-g]thieno[2′,3′:4,5]thieno[3,2-b]indole-2,10-diyl)bis(methaneylylidene))bis(5,6-difluoro-3-oxo-2,3-dihydro-1H-indene-2,1-diylidene))dimalononitrile (BSeC8-4F) was synthesized as shown in Scheme S1. All intermediates and the final product were fully characterized by ^1^H and ^13^C NMR and elementary analysis (Figs. S30-S37). The polymer 1-(5-(4,8-bis(5-(2-ethylhexyl)-4-fluorothiophen-2-yl)-6-methylbenzo[1,2-b:4,5-b′]dithiophen-2-yl)thiophen-2-yl)-5,7-bis(2-ethylhexyl)-3-(5-methylthiophen-2-yl)-4H,8H-benzo[1,2-c:4,5-c′]dithiophene-4,8-dione (PM6) was selected as the donor to couple with BSeC8-4F due to their complementary absorption from 300 to 1000 nm (Fig. 1b). The PM6 film exhibited an absorption band at 420–700 nm, with a peak at 623 nm and a shoulder at 583 nm, while BSeC8-4F film possessed a broad absorption in the range from 570 to 1000 nm with a strong absorption peak at 850 nm (Fig. 1b). The optical bandgaps of BSeC8-4F and PM6 were estimated to be 1.32 and 1.81 eV, respectively, according to the onset of the optical absorption spectra. The ultraviolet photoelectron spectroscopy (UPS) was employed to measure the accurate energy levels of PM6 and BSeC8-4F (Fig. 1c–d). The highest occupied molecular orbital (HOMO) energy levels were estimated to be − 5.50 and − 5.71 eV for PM6 and BSeC8-4F, respectively, while the lowest unoccupied molecular orbital (LUMO) energy levels of PM6 (− 3.69 eV) and BSeC8-4F (− 4.39 eV) were calculated based on the optical bandgaps and HOMO levels. As shown in Fig. S1a, thermogravimetric analysis (TGA) showed that BSeC8-4F exhibited good thermal stability with decomposition temperatures (5% weight loss) up to 344 °C. In differential scanning calorimetry (DSC) spectra (Fig. S1b), BSeC8-4F exhibited an exothermal peak at 225 °C, corresponding to the crystallization temperature (*T*_c_). The photoluminescence (PL) spectra of the PM6:BSeC8-4F film revealed that the fluorescence intensities of the donor and acceptor were effectively quenched, indicating an efficient charge transfer between the donor and acceptor (Fig. S1c).

### Organic Optoelectronic Synaptic Devices

**Fig. 2:**
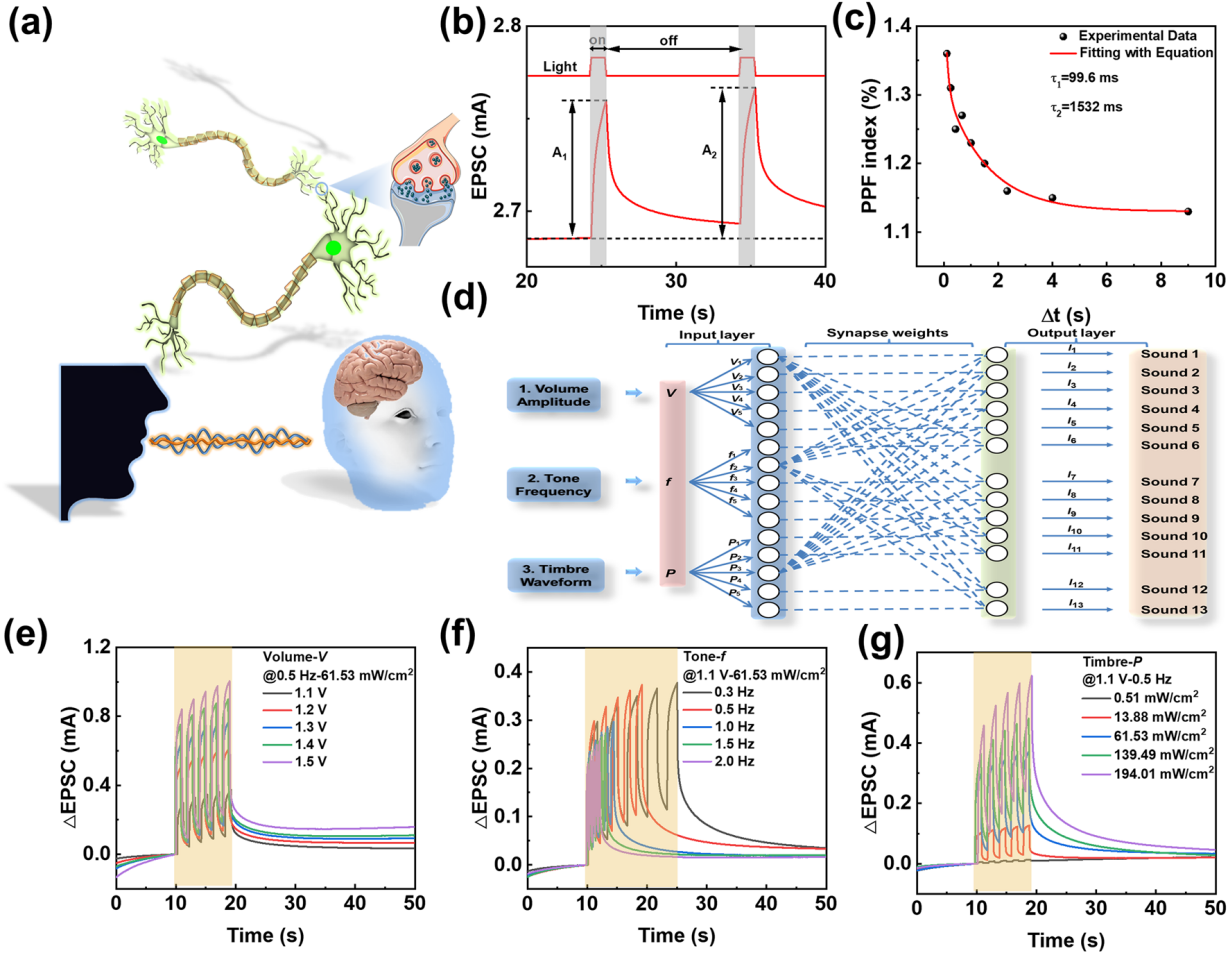
**a** Schematic images of biological synapse in neural system of human brain. **b** Typical photo-responsive characteristic of OOSs. **c** The variation of PPF index with the interval of light pulse pairs. **d** Schematic images of sound perception based on OOSs. **e**–**g** The volume, tone and timbre of sound simulated by input signal of voltage **e**, frequency **f** and light intensity **g** at 850 nm

OOSs with a device structure of ITO/PEDOT:PSS/D:A/PDINN/Ag were fabricated (Fig. 1a), while the schematic image of the biological synapse in neural system of human brain was shown in Fig. 2a. The typical photo-responsive characteristics of OOS at forward bias were investigated upon a series of light pulse pairs with varied interval (Δt) values, wherein all pulses had a width of 1 s and an intensity of 61.53 mW cm^−2^ (Figs. 2b and S2). Paired-pulse facilitation (PPF) defined by: PPF = A_2_/A_1_ × 100%, described the fact that a second pre-synaptic spike caused an enhanced post-synaptic current than the first one, which was essential to recognize and decode temporal information such as visual and auditory signals in a biological neural system [23]. The PPF index that described the short-term plasticity (STP) of synapse could be fitted by a double exponential function:

1 $${\text{PPF}} = 1 + {\text{C}}_{1} \times \exp ( - \Delta {\text{T}}/\tau_{1} ) + {\text{C}}_{2} \times \exp ( - \Delta {\text{T}}/\tau_{2} )$$

where C_1_ and C_2_ represented the initial rapid and slow phase facilitation magnitudes, and *τ*_1_ and *τ*_2_ were the characteristic relaxation times of these phases, respectively. Device I exhibited PPF index with *τ*_1_ and *τ*_2_ of 99.6 and 1532 ms, respectively (Fig. 2c), which was similar to those in biological synapses [24].

The photo-responsive characteristics of the device depends on the built-in potential (φ) and applied external bias. The built-in potential (φ = 0.826 V) could be obtained by measuring the photovoltaic performance of Device I (Fig. S3) [25–27]. When the applied voltage was lower than the built-in potential, the device presented fast response to the photo excitation, while the synapses characteristics with excitatory postsynaptic current (EPSC) appeared when the applied bias (0.9–1.5 V) exceeded the built in potential (Fig. S4). The spike rate, spike light intensity, spike number and spike duration correspond to the learning rules of spike-rate-dependent plasticity (SRDP), spike-light intensity-dependent plasticity (SIDP), spike-number dependent plasticity (SNDP) and spike-duration-dependent plasticity (SDDP), respectively. In a biological synapse, the synaptic weight can be modified by controlling the fire rate of the presynaptic spikes [28]. Thus, a low frequency training resulted in long-term plasticity (LTP) (Fig. S5). With the increase of light intensity, stimulation times and duration of illumination time, the short-term plasticity (STP) changed to LTP (Figs. S6–S8), which is in accordance with the transition of short-term memory (STM) to long-term memory (LTM) in human brains [29, 30]. In the pulse light stimulating process (Fig. S9), the time of photocurrent up to 2.78 mA continuously decreased from the first (4.30 s) to the tenth (0.30 s) exposure, similar to the learning behavior of human brains [23]. Simultaneously, the device exhibits excellent repeatability and stability (Fig. S10).

### Sound Perception

The function of the OOS for sound perception via adjusting the input signal including voltages, frequencies and light intensities was investigated (Fig. 2d). The essential factors of sound including volume, tone, and timbre are affected by amplitude, frequency, and waveform of sound wave, respectively. Specifically, the size of postsynaptic current (*I* = *I*_light _− *I*_dark_) changed with the input voltage, corresponding to the variation characteristics of sound amplitude (Fig. 2e). Moreover, the variable speed of synaptic signal changed with the input frequency of the device, which was consistent with the variation of sound frequency (Fig. 2f). Also, the shape of synaptic signal changed with light intensity on the device, equivalent to the variation of sound waveform (Fig. 2g). Thus, the volume, tone and timbre of sound can be simulated appropriately by tuning the input signal of voltages, frequencies and light intensities of OOS.

The sounds with various volume, tone and timbre were collected by knocking a capped glass bottle with water inside (Figs. S11–S16). The amplitude changes of the sounds were produced by altering the vertical height of the paper clip (10, 15, 20, 25 and 30 cm) on the water (80 mL) contained glass bottle without bottle cap, which were simulated by varying input voltage (1.1, 1.2, 1.3, 1.4 and 1.5 V) (Fig. S11), and the corresponding sound waves and Fourier transforms (FTs) were showed in Fig. S12. Also, the frequency changes of sounds that were simulated by changing input frequencies (0.3, 0.5, 1.0, 1.5 and 2.0 Hz) were collected by altering the water levels (100, 80, 60, 40 and 20 mL), while the vertical height (10 cm) were kept the same for the bottle without cap (Fig. S13). The corresponding sound waves and FTs were shown in Fig. S14. Moreover, the waveform changes of sounds, simulated by altering the light intensities (0.51, 13.88, 61.53, 139.49 and 194.01 mW cm^−2^), were achieved by altering the glass bottles with different caps while the vertical height (10 cm) and water content (80 mL) stayed the same (Fig. S15). The corresponding sound waves and FTs were presented in Fig. S16. In addition, a random sound was produced at 25 cm of vertical height and 120 mL of water without the gap, and the corresponding input conditions of the device were 1.4 V, 0.2 Hz and 61.53 mW cm^−2^ (Fig. S17).

**Fig. 3:**
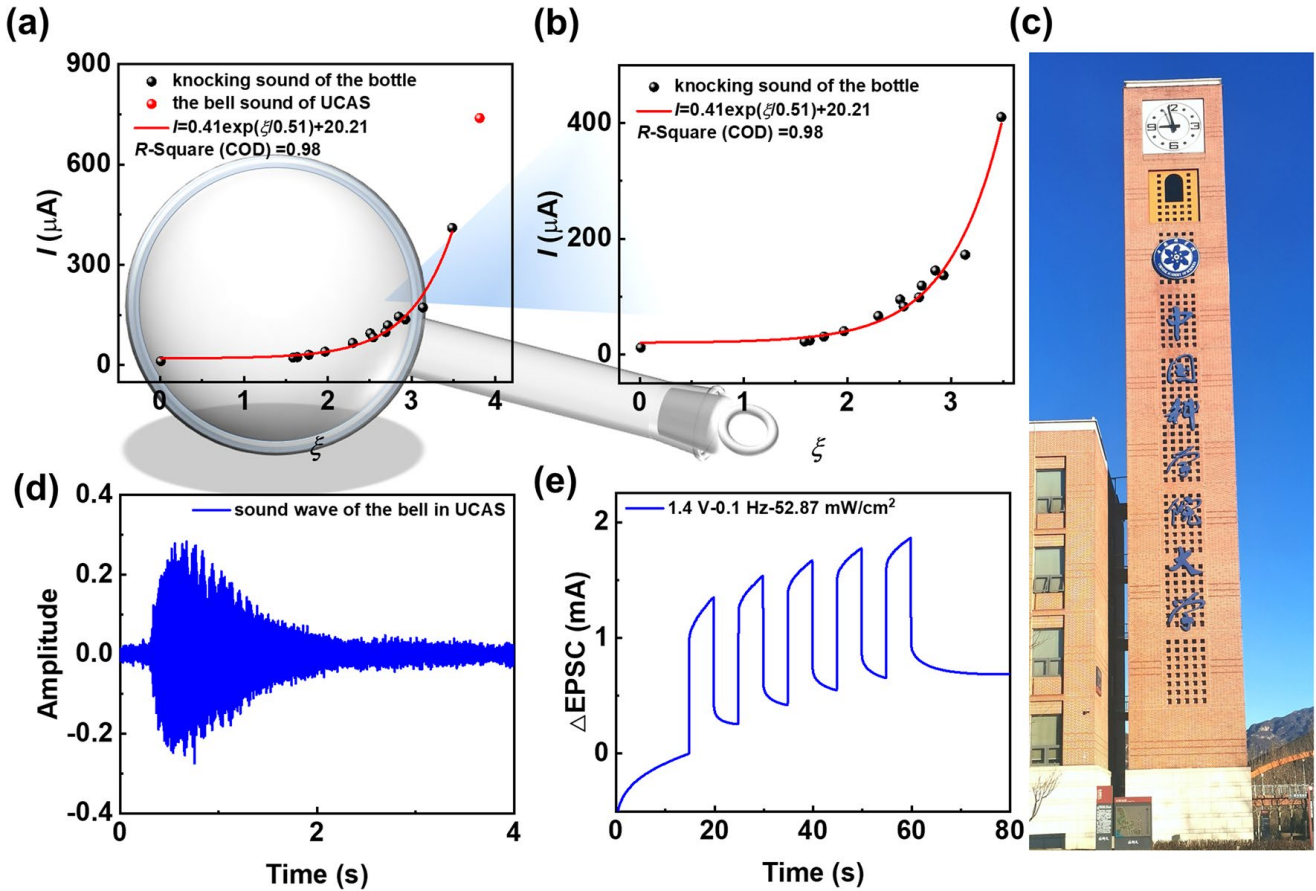
**a**–**b** Quantitative relationship of postsynaptic current (*I*) and perception factor (*ζ*) for sound perception based on Device I, and (**b**) partial enlarged view of **a**. **c** Bell tower of the UCAS. **d** Sound wave of the bell. **e** The corresponding synaptic signal of the bell sound

The series of sounds with different input conditions possessed various excitatory postsynaptic currents (EPSC). To identify different EPSCs for sound perception, a novel parameter named recognition factor (*ζ*) was proposed. The equation of *ζ* can be represented as:

2 $$\zeta = V\log \frac{p}{f}$$

where *V*, *f* and *P* are the input voltage, input frequency and light intensity on device, respectively. The value of *ζ* and the postsynaptic current (*I*) after removing the light for 5 s is shown in Table 1. The quantitative relation between *ζ* and *I* was established (Fig. 3a–b), which could be expressed as:

3 $$I = 0.41\exp \left( {\zeta /0.51} \right) \, + \, 20.21$$

**Table 1:** The modulation of sound signal via the input signal of voltage, frequency and light intensity of OOS based on PM6:BSeC8-4F system

Voltage (V)	Frequency (Hz)	Light intensity (mW cm^−2^)	*ζ*	*I*^a^ (μA)	Definition
Volume (amplitude)	Tone (frequency)	Timbre (waveform)			
1.1	0.5	61.53	2.30	66.52	Sound 1
1.2	0.5	61.53	2.51	95.10	Sound 2
1.3	0.5	61.53	2.72	119.10	Sound 3
1.4	0.5	61.53	2.93	136.80	Sound 4
1.5	0.5	61.53	3.14	172.50	Sound 5
1.1	0.3	61.53	2.54	83.17	Sound 6
1.1	1.0	61.53	1.97	40.35	Sound 7
1.1	1.5	61.53	1.77	30.93	Sound 8
1.1	2.0	61.53	1.64	24.04	Sound 9
1.1	0.5	0.51	0.00946	12.01	Sound 10
1.1	0.5	13.88	1.59	22.59	Sound 11
1.1	0.5	139.49	2.70	98.85	Sound 12
1.1	0.5	194.01	2.85	145.02	Sound 13
1.4	0.2	61.53	3.48	410.50	Sound 14
1.4	0.1	52.87	3.81	738.90^b^	Bell sound
1.4	0.1	52.87	3.81	740.02^c^	Bell sound

^a^The postsynaptic current (*I*) after removing the light for 5 s

^b^The experimental value of bell sound, which was test by the equipment under the input condition of 1.4 V, 0.1 Hz and 52.87 mW cm^−2^

^c^The theoretical value of bell sound according to Eq. (3)

*R*-Square (COD) = 0.98.

Therefore, the sounds corresponding specific input conditions (*V*, f, *P*) could stimulate OOSs to produce different postsynaptic currents (experimental value, *I*_exper_). According to Eq. (3), the *ζ* value corresponds to a theoretical value (*I*_theo_). When the value of *I*_theo_ is approximately equal to that of *I*_exper_, the corresponding *ζ* can be determined. Thus, the sounds with specific *ζ* can be perceived. In practice, the sound of the bell tower of University of Chinese Academy of Sciences (UCAS) was recorded and simulated as shown in Fig. 3c–e and Fig. S18. The sound wave of the bell (Fig. 3d) corresponded to the synaptic signal (Fig. 3e) at 1.4 V, 0.1 Hz and 52.87 mW cm^−2^. Thus, the *I*_exper_ and *I*_theo_ of bell sound were 738.90 and 740.02 μA, respectively, with accuracy of 99.8%, which was sufficient to perceive the bell sound.

The universality of the sound perception function for these organic optoelectronic synapses were investigated through varying the donor and acceptor materials (Figs. S19–S20). The quantitative relationships of postsynaptic current (*I*) and recognition factor (*ζ*) for sound perception based on PBDB-T:ITIC and P3HT:PC_71_BM systems were shown in Figs. S21–S22. The values (Table S1) showed that the sound perception was achieved in different systems based on the implementation of *ζ* and *I*, revealing the generality of this working mechanism for sound perception.

### Mechanism Studies

**Fig. 4:**
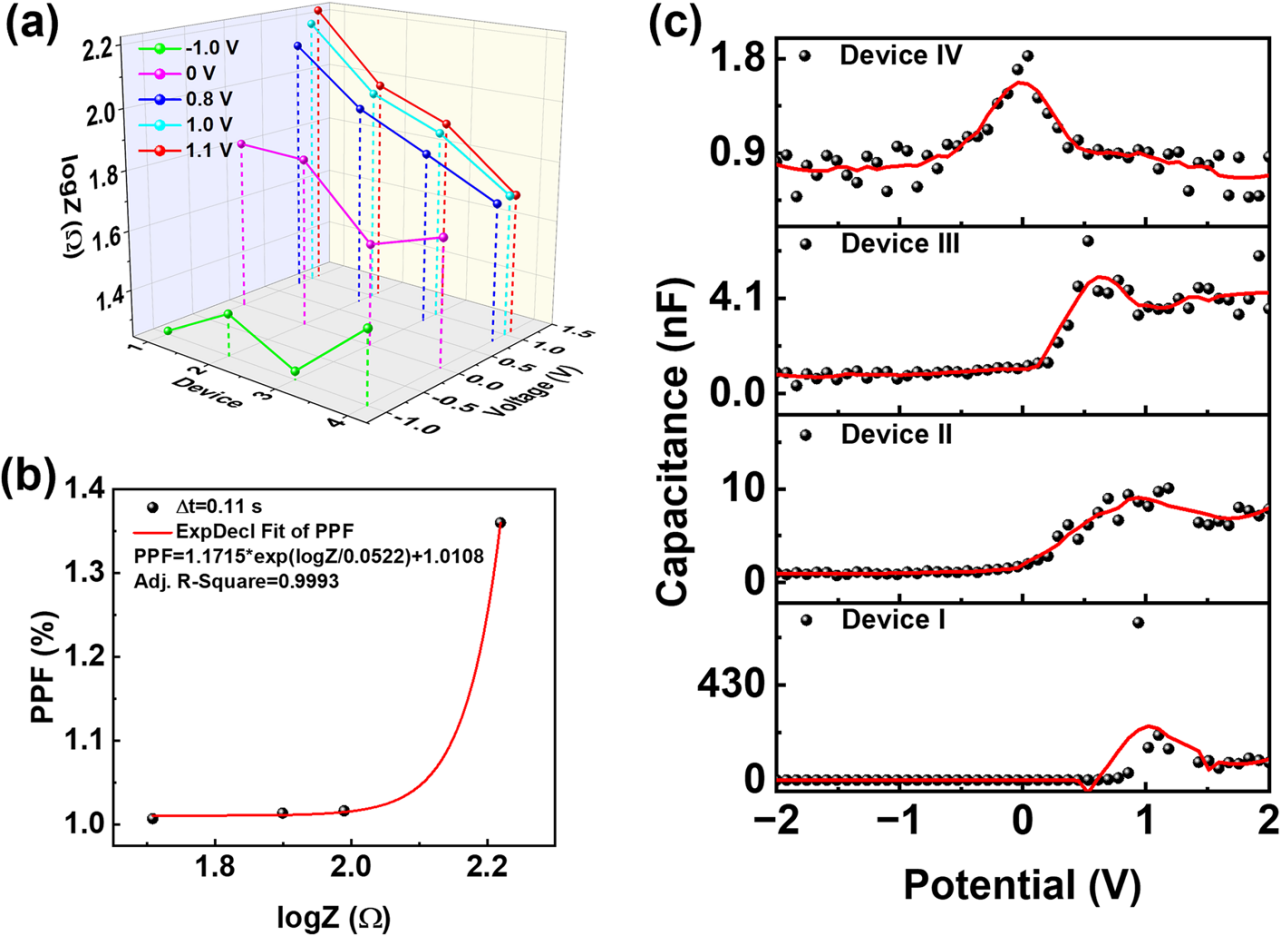
**a** Impedances (logZ) of Device I (ITO/PEDOT:PSS/D:A/PDINN/Ag), Device II (ITO/PEDOT:PSS/D:A/Ag), Device III (ITO/D:A/PDINN/Ag) and Device IV (ITO/D:A/Ag) under different bias voltages. **b** Quantitative relationship curve of PPF index versus logZ plots at the light pulse pairs with interval of (Δt) 0.11 s. **c** The capacitance–voltage (C–V) profile of Device I, Device II, Device III and Device IV at 850 nm light

To investigate the functions of the interfacial layers, four types of devices with different structures of ITO/PEDOT:PSS/D:A/PDINN/Ag (Device I), ITO/PEDOT:PSS/D:A/Ag (Device II), ITO/D:A/PDINN/Ag (Device III), and ITO/D:A/Ag (Device IV) were fabricated (Fig. S23). Under positive bias voltage (> *V*_oc_), the electrons transport to the ITO electrode by the hole transport layer (HTL), and hole transport to the metal electrode by the electron transport layer (ETL), resulting in the occurrence of charge trapping at interface layer for Device I. Obvious synaptic signals were observed in Device I, while slight synaptic signals were presented in Device II and Device III (Figs. 2e–g and S24–S25). However, almost no synaptic signal was observed in Device IV (Fig. S26). Thus, the interfacial layers (PEDOT:PSS and PDINN) are critical for the generation of synaptic signals. To further study the role of interfacial layers on the synaptic performances, the impedance-voltage (*Z*–*V*) profile was implemented to investigate the interface resistances of the Device I-IV under different bias voltages (Figs. 4a and S27, Table S2). With the increase of bias voltage, the interface resistances (*R*_int_) continuously improved, which may lead to the charge trapping at interface layer. Under the same bias (forward bias), the interface resistance of the devices followed the order of Device I > Device II > Device III > Device IV. Moreover, the quantitative curves of PPF index versus logZ plots at the light pulse pairs with various interval values were presented, which revealed that the PPF indexes increased along with the enhancement of the interface resistances (Figs. 4b and S28–S29). Thus, with the simultaneous existence of electron blocking layer (EBL) and hole blocking layer (HBL), Device I possessed the maximum interface resistance among the four devices at forward bias, which is accordant with the highest PPF value of Device I. In addition, capacitance–voltage (C–V) profile was measured to probe the mechanism (Fig. 4c). The Device I possessed the maximum peak voltage due to its maximum interface barrier, leading to utmost interface resistance. With the decrease of charge trap ability, the peak voltages changed with the order of Device I (0.94 V) > Device II (0.80 V) > Device III (0.53 V) > Device IV (0.04 V), in accordance to the change of interface resistance.

## Conclusions

The OOSs were constructed for unprecedented sound perception upon tuning the interfacial layers. Impressively, the volume, tone and timbre of sound influenced by the amplitude, frequency and waveform were simulated appropriately by the input signal of voltages, frequencies and light intensities of OOSs. The quantitative relation between recognition factor (*ζ*) and postsynaptic current (*I* = *I*_light _− *I*_dark_) was established to achieve highly accurate sound perception. The mechanistic studies revealed the impedance of the interfacial layer are critical in the synaptic performances. This contribution paves the way for the development of OOSs in the artificial intelligence.

### Electronic supplementary material

Below is the link to the electronic supplementary material.

Supplementary Material 1
