# Supplementary material for: Organic Optoelectronic Synapses for Sound Perception
Source: Nanomicro Lett. 2023 May 24;15:133. doi: 10.1007/s40820-023-01116-3 (PMC10205940; doi:10.1007/s40820-023-01116-3)
Supplement: Supplementary file 1 — Supplementary Material 1 [file 40820_2023_1116_MOESM1_ESM.pdf]

Supporting Information for

Organic Optoelectronic Synapses for Sound Perception

Yanan Wei[1], Youxing Liu[2], Qijie Lin[1], Tianhua Liu[1], Song Wang[1], Hao Chen[1], Congqi Li[1], Xiaobin Gu[1], Xin Zhang[1, *], Hui Huang[1, *]

[1]College of Materials Science and Opto-Electronic Technology & Center of Materials Science and Optoelectronics Engineering & CAS Center for Excellence in Topological Quantum Computation & CAS Key Laboratory of Vacuum Physic, University of Chinese Academy of Sciences, Beijing 100049, P. R. China

[2]School of Materials Science and Engineering, Peking University, Beijing 100871, P. R. China

*Corresponding authors. E-mail: huihuang@ucas.ac.cn (Hui Huang), zhangxin2019@ucas.ac.cn (Xin Zhang)

S1 Experimental Section

S1.1 Sound Collections

The sound with various volumes, tones and timbres were collected by knocking a capped glass bottle with water inside. First, the sounds with different volumes were collected by changing the vertical height of the paper clip. Thus, the amplitude changes of the sounds were produced via altering the vertical height of 10, 15, 20, 25 and 30 cm, which were based on water (80 mL) without cap. Second, the sounds with different tones were collected by changing water level. Thus, the frequency changes of sounds were produced by altering the water levels of 100, 80, 60, 40 and 20 mL, which were based on the same vertical height (10 cm) without cap. Third, the sounds with different timbres were collected by changing the structure of the bottle. Thus, the waveform changes of sounds were achieved by altering the glass bottles with bottle cap of parafilm, dark, none, red and yellow, while the vertical height (10 cm) and water content (80 mL) were kept the same. In addition, a random sound is produced at 25 cm of vertical height and 120 mL of water without the gap. In practice, the bell sound of University of Chinese Academy of Sciences (UCAS) was collected by a mobile phone.

S1.2 Sound Modulation by Organic Optoelectronic Synapse

With the change of input voltage, frequency and light intensity, the size, speed and shape of synaptic signal were tuned, respectively, which correspond to the amplitude, frequency, and waveform of sound wave, thus achieving the modulation of volume, tone and timbre of sound. The amplitude changes can be simulated by input voltage (1.1, 1.2, 1.3, 1.4 and 1.5 V), which is consistent with volume modulation of sound. The frequency changes can be simulated by input frequency (0.3, 0.5, 1.0, 1.5 and 2.0 Hz), corresponding to the frequency modulation of

sounds. The waveform changes of sounds can be simulated by altering the input light intensities (0.51, 13.88, 61.53, 139.49 and 194.01 mW/cm^2), equivalent to the timbre modulation of sounds. And the input conditions of random sound at vertical height (25 cm) and water (120 cm) without the gap is 1.4 V, 0.2 Hz and 61.53 mW/cm^2.

In addition, for the same amplitude of the bell sound (UCAS) and sound 4 (25 cm-80 mL-none), the amplitude of the bell sound was simulated by input voltage of 1.4 V. The frequency was one fifth of that of sound 4 (25 cm-80 mL-none). Thus, the input frequency was 0.1 Hz. For the different timber of sound, the input light intensity was 52.87 mW/cm^2. Thus, the bell sound was simulated under the input condition of 1.4 V, 0.1 Hz, and 52.87 mW/cm^2

S2 Supporting Scheme

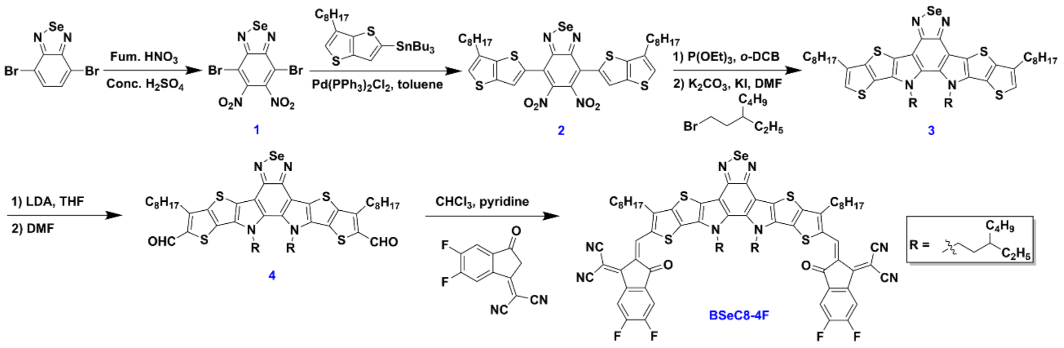

Scheme S1 The synthetic route of **BSeC8-4F**

S3 Synthetic Section

The compound 4,7-dibromobenzo[*c*][1,2,5]selenadiazole was synthesized according to the previous literature.[S1]

Synthesis of 4,7-dibromo-5,6-dinitrobenzo[c][1,2,5]selenadiazole (1)

The compound 4,7-dibromobenzo[*c*][1,2,5]selenadiazole (3.41 g, 10.00 mmol) was added with small portions into a mixture of sulfuric acid and fuming nitric acid (1:1, 50 mL) under stirring at 0 °C over 20 min. After stirring at room temperature overnight, the mixture was poured into ice water slowly. The precipitate was collected by filtration and washed with water, dried under vacuum to afford the compound **1** (2.89 g, 6.71 mmol, 67.1%), which was used directly without further purification. ^{13}C NMR (126 MHz, $CDCl_3$, δ): 154.93, 144.89, 112.57 ppm.

Synthesis of 5,6-dinitro-4,7-bis(6-octylthieno[3,2-b]thiophen-2-yl)benzo[c][1,2,5]selenadiazole (2)

The compound **1** (861.7 mg, 2.00 mmol) and tributyl(6-octylthieno[3,2-*b*]thiophen-2-yl)stannane (2.44 g, 4.50 mmol) were dissolved in dry toluene (25.0 mL), which was deoxygenated with nitrogen for 10 min. $Pd(PPh_3)_2Cl_2$ (140.4 mg, 0.20 mmol) was then added under nitrogen, and the reaction was stirred at 80 °C overnight. After cooled to room

temperature, the mixture was poured into water and extracted with dichloromethane (100 mL × 3 times). The organic phase was combined and washed with water and dried over anhydrous Na_2SO_4. Afterwards, the solution was filtered, and concentrated under reduced pressure. The crude product was purified via column chromatography on silica gel (petroleum ether/DCM, 3:1) to afford a purple solid (1.06 g, 1.37 mmol, 68.5%). ^{1}H NMR (500 MHz, $CDCl_3$, δ): 7.64 (s, 2H), 7.16 (s, 2H), 2.77 (t, J = 7.7 Hz, 4H), 1.78 (p, J = 7.6 Hz, 4H), 1.44–1.24 (m, 20H), 0.92–0.84 (m, 6H); ^{13}C NMR (126 MHz, $CDCl_3$, δ): 156.87, 144.40, 142.14, 139.00, 135.18, 130.73, 125.07, 124.04, 122.70, 32.00, 29.96, 29.53, 29.47, 29.37, 28.69, 22.81, 14.27.

Synthesis of 12,13-bis(3-ethylheptyl)-3,9-dioctyl-12,13-dihydro-[1,2,5]selenadiazolo[3,4-e]thieno[2'',3'':4',5']thieno[2',3':4,5]pyrrolo[3,2-g]thieno[2',3':4,5]thieno[3,2-b]indole (3)

The compound 5,6-dinitro-4,7-bis(6-octylthieno[3,2-*b*]thiophen-2-yl)benzo[*c*][1,2,5]selenadiazole **(2)** (773.9 mg, 1.00 mmol) and triethyl phosphite (2.0 mL) were dissolved in the *o*-dichlorobenzene (15.0 mL). The resulting mixture was refluxed for 24 hours under nitrogen. After removal of the solvent under reduced pressure, the red residue was obtained, which was directly used in the next reaction.

The compounds 1-bromo-3-ethylheptane (1.24 g, 6.00 mmol), potassium carbonate (2.07 g, 15.00 mmol), potassium iodide (16.6 mg, 0.10 mmol), and the red residue from above reaction were added into DMF (20.0 mL). The mixture was refluxed at 90 °C for 12 h. After cooled to room temperature, the mixture was poured into water and extracted with dichloromethane (DCM) for three times. The combined organic phase was washed with water and dried over anhydrous Na_2SO_4. Afterwards, the solution was filtered, and concentrated under reduced pressure. The crude product was purified via column chromatography on silica gel (petroleum ether/DCM, 5:1) to afford a red solid (396.5 mg, 0.41 mmol, 41.2%). ^{1}H NMR (500 MHz, $CDCl_3$, δ): 6.99 (s, 2H), 4.69–4.59 (m, 4H), 2.85–2.77 (m, 4H), 1.88–1.71 (m, 8H), 1.46–1.13 (m, 30H), 1.10–0.86 (m, 14H), 0.71–0.63 (m, 12H); ^{13}C NMR (126 MHz, $CDCl_3$, δ): 153.79, 142.22, 137.08, 136.99, 131.26, 123.91, 123.47, 119.20, 113.97, 49.51, 36.99, 34.25, 32.77, 32.04, 29.79, 29.63, 29.57, 29.43, 28.96, 28.85, 26.05, 23.04, 22.82, 14.27, 14.11, 10.86.

Synthesis of 12,13-bis(3-ethylheptyl)-3,9-dioctyl-12,13-dihydro-[1,2,5]selenadiazolo[3,4-e]thieno[2'',3'':4',5']thieno[2',3':4,5]pyrrolo[3,2-g]thieno[2',3':4,5]thieno[3,2-b]indole-2,10-dicarbaldehyde (4)

To an anhydrous THF (20.0 mL) solution of compound **3** (362.7 mg, 0.30 mmol), LDA (2.0 M in THF/hexane, 0.60 mL, 1.20 mmol) was added dropwise under the protection of argon at –78 °C. After the mixture was stirred at –78 °C for 1 h, DMF (1.0 mL) was added. Then the mixture was warmed to room temperature and stirred overnight. The mixture was poured into water and extracted with DCM (50 mL × 3). The combined organic layer was dried over anhydrous Na_2SO_4. Afterwards, the solution was filtered, and evaporated to afforded a orange solid, which was purified via column chromatography on silica gel (petroleum ether/CH_2Cl_2, 1:2) to afford a red solid (211.7 mg, 0.21 mmol, 69.3%). ^{1}H NMR (500 MHz, $CDCl_3$, δ): 10.13

(s, 2H), 4.75–4.63 (m, 4H), 3.24–3.15 (m, 4H), 1.96–1.68 (m, 8H), 1.50–1.10 (m, 30H), 1.07–0.84 (m, 14H), 0.67–0.61 (m, 12H); ^{13}C NMR (126 MHz, $CDCl_3$, δ): 181.80, 153.24, 146.90, 143.28, 136.93, 136.66, 132.60, 129.43, 128.56, 114.77, 49.75, 37.00, 34.46, 32.68, 31.95, 30.43, 29.75, 29.46, 29.32, 28.85, 28.24, 25.98, 22.98, 22.77, 14.22, 14.07, 10.85.

Synthesis of BSeC8-4F

To a $CHCl_3$ (10.0 mL) solution of compound **4** (101.8 mg, 0.10 mmol), 2-(5,6-difluoro-3-oxo-2,3-dihydro-1*H*-inden-1-ylidene)malononitrile (92.1 mg, 0.40 mmol) and pyridine (0.20 mL) in $CHCl_3$ (20.0 mL) were added, which was heated at 60°C overnight. After cooling to room temperature, the mixture was concentrated to 5 mL and poured into methanol (50.0 mL) and then filtered. The residue was purified via column chromatography on silica gel with chloroform as the eluent to afford a dark blue solid (125.5 mg, 0.087 mmol, 87.0%). ^{1}H NMR (500 MHz, $CDCl_3$, δ): 8.72 (s, 2H), 8.39 (dd, J = 9.9, 6.4 Hz, 2H), 7.58 (t, J = 7.5 Hz, 2H), 4.76–4.58 (m, 4H), 3.03–2.86 (m, 4H), 1.97–1.68 (m, 8H), 1.46–1.04 (m, 38H), 0.90–0.85 (m, 6H), 0.84–0.72 (m, 12H); MS (MALDI-TOF): *m/z* calcd. for $C_{78}H_{78}F_4N_8O_2S_4Se$: 1442.42; found: 1442.13.

S4 Supplementary Figures

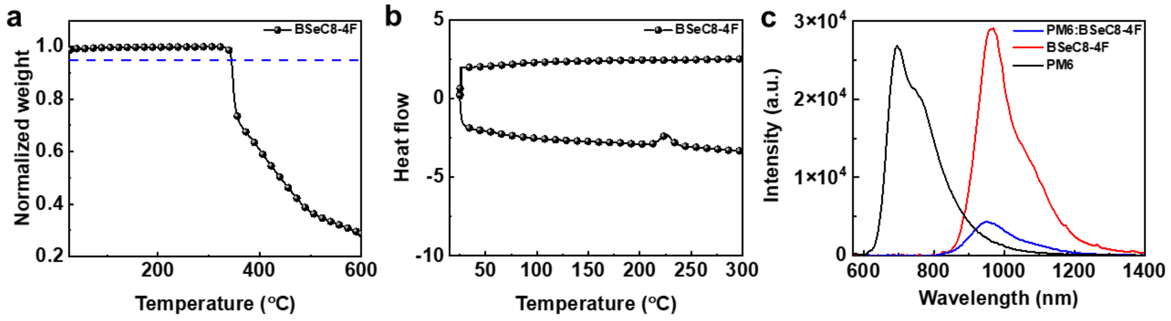

Fig. S1 a, TGA curve and **b**, DSC curve for BSeC8-4F. **c**, PL measurements of PM6, BSeC8-4F and PM6:BSeC8-4F

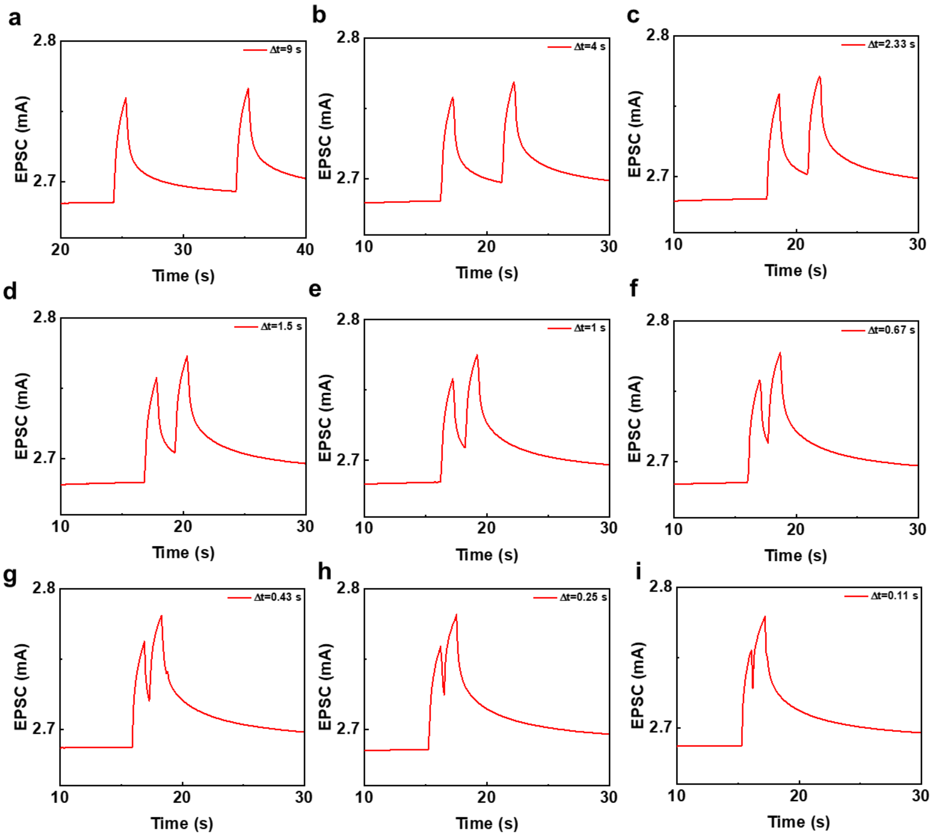

Fig. S2 EPSC values based on light pulse paired with varied interval (Δt) of Device I at 850 nm

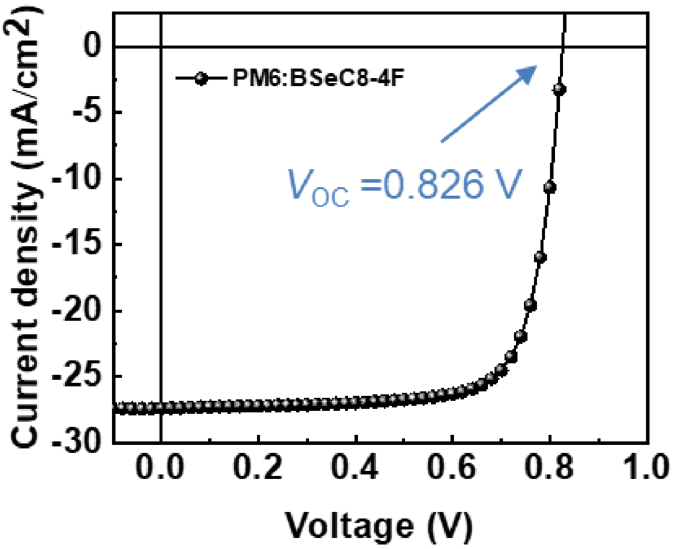

Fig. S3 Built-in potential of Device I obtained by measuring the open circuit voltage (V_{oc}) of corresponding organic solar cells

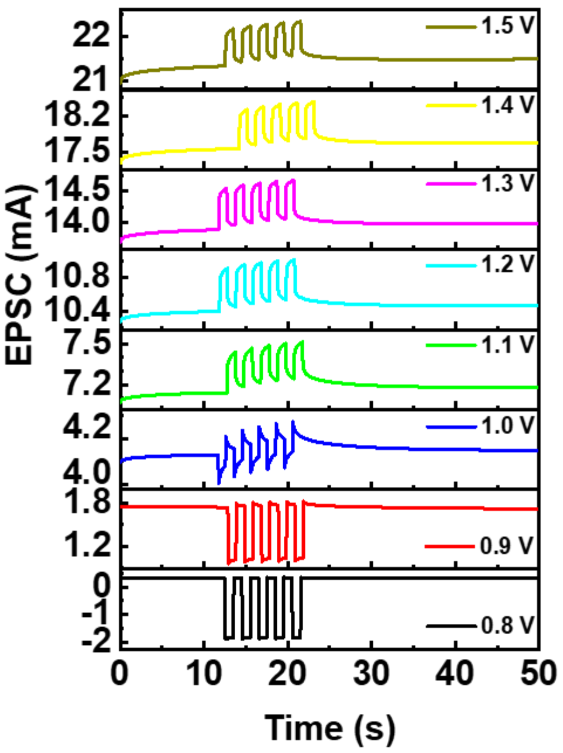

Fig. S4 Spike-voltage-dependent plasticity (SVDP) of Device I based on PM6:BSeC8-4F film at 850 nm

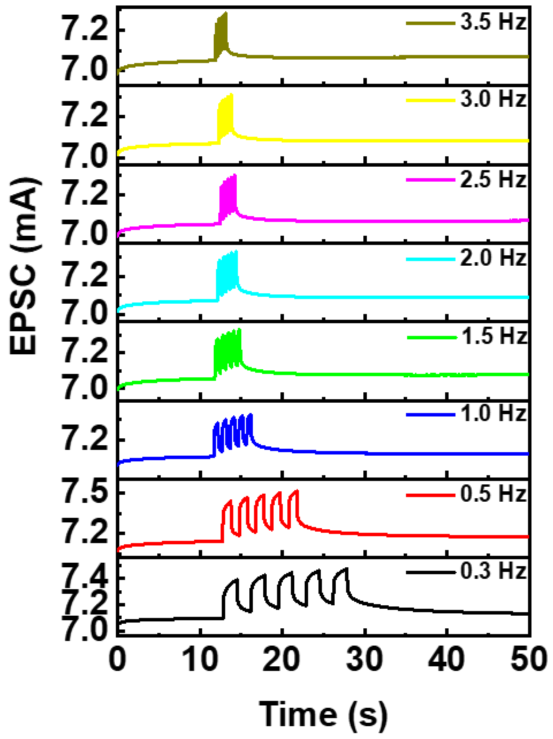

Fig. S5 Spike-rate-dependent plasticity (SRDP) of Device I based on PM6:BSeC8-4F film at 850 nm

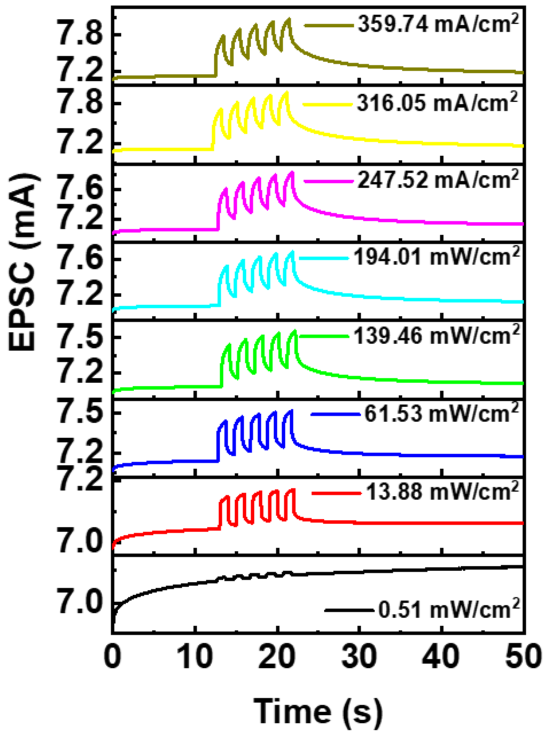

Fig. S6 Spike-light-intensity dependent plasticity (SIDP) of Device I based on PM6:BSeC8-4F film at 850 nm

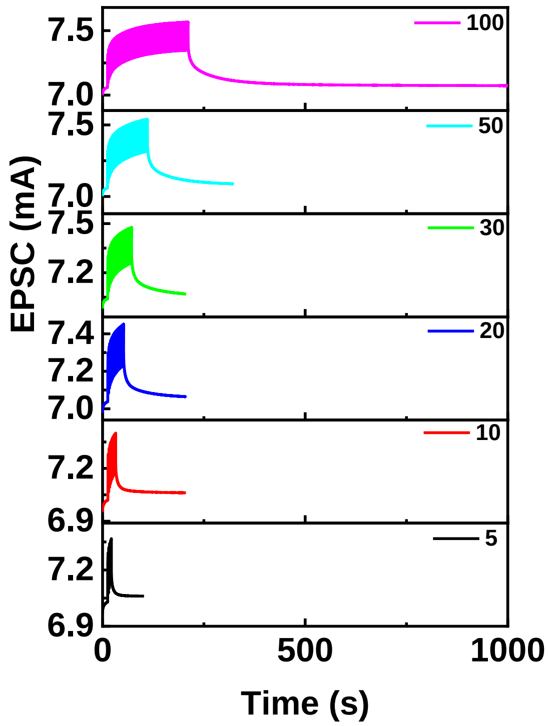

Fig. S7 Spike-number-dependent plasticity (SNDP) of Device I based on PM6:BSeC8-4F film at 850 nm

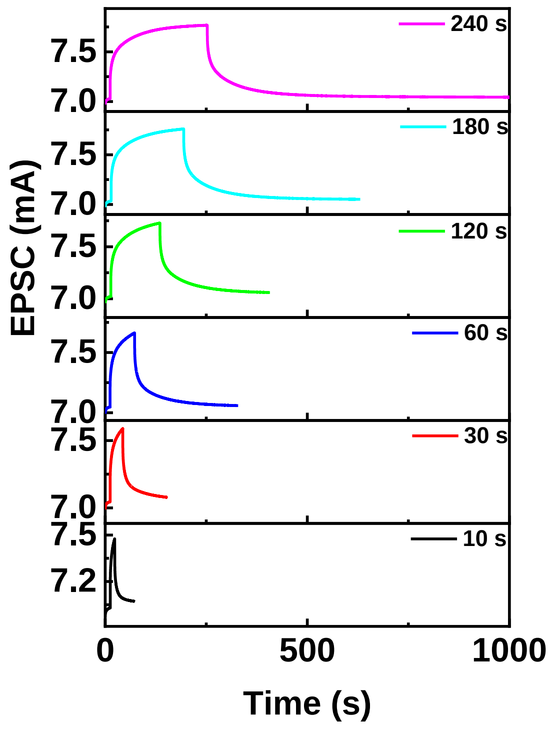

Fig. S8 Spike-duration-dependent plasticity (SDDP) of Device I based on PM6:BSeC8-4F film at 850 nm

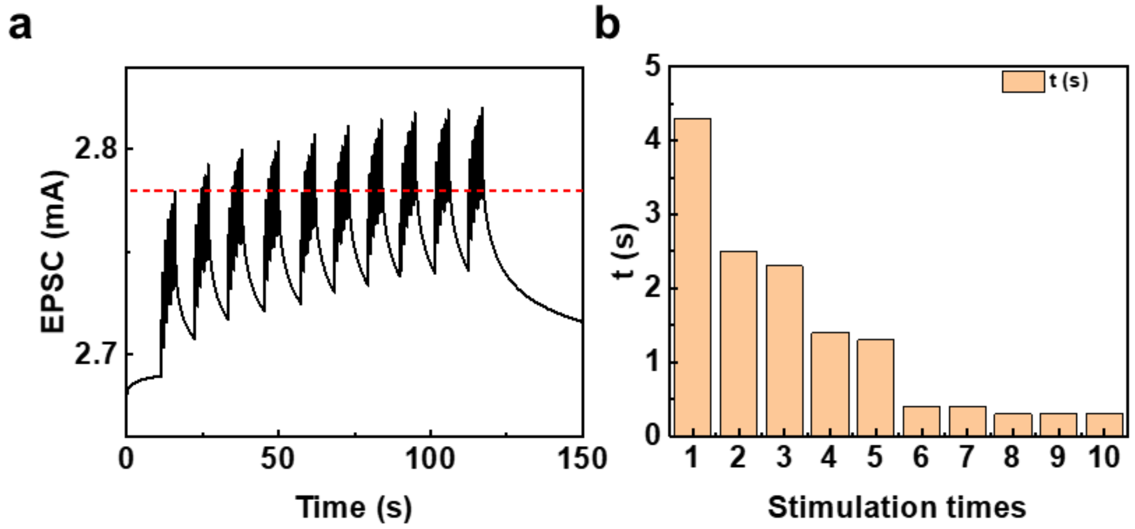

Fig. S9 The "learning-experience" behavior under pulsed light stimuli (850 nm, 61.53 mW/cm^2)

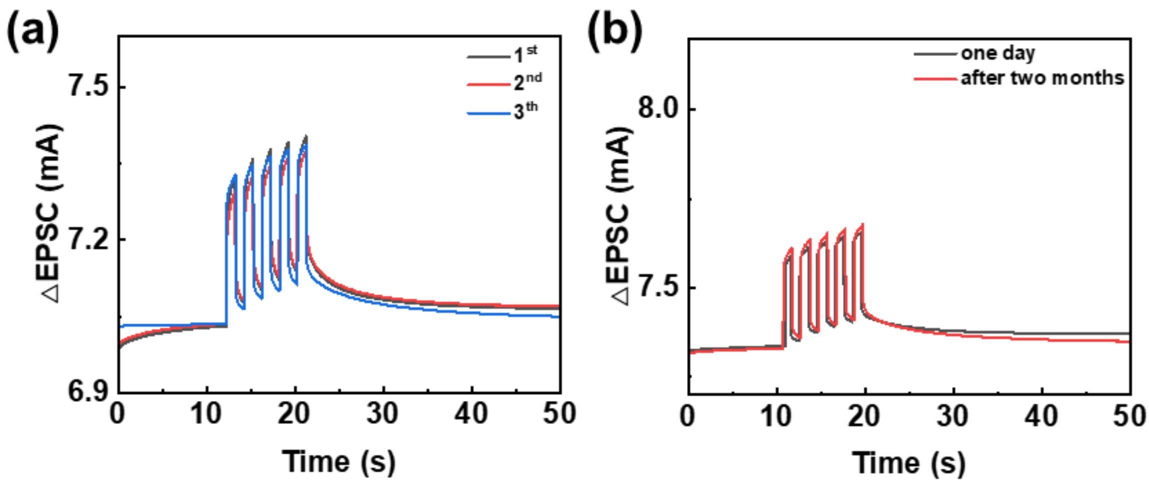

Fig. S10 The repeatability and stability of Device I in ambient conditions

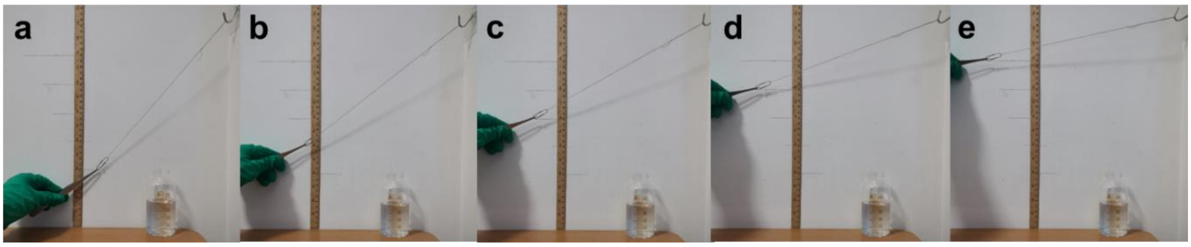

Fig. S11 The sounds with various volumes. Falling height of **a**, 10 cm, **b**, 15 cm, **c**, 20 cm, **d**, 25 cm and **e**, 30 cm on glass bottles with water (80 mL) without bottle cap

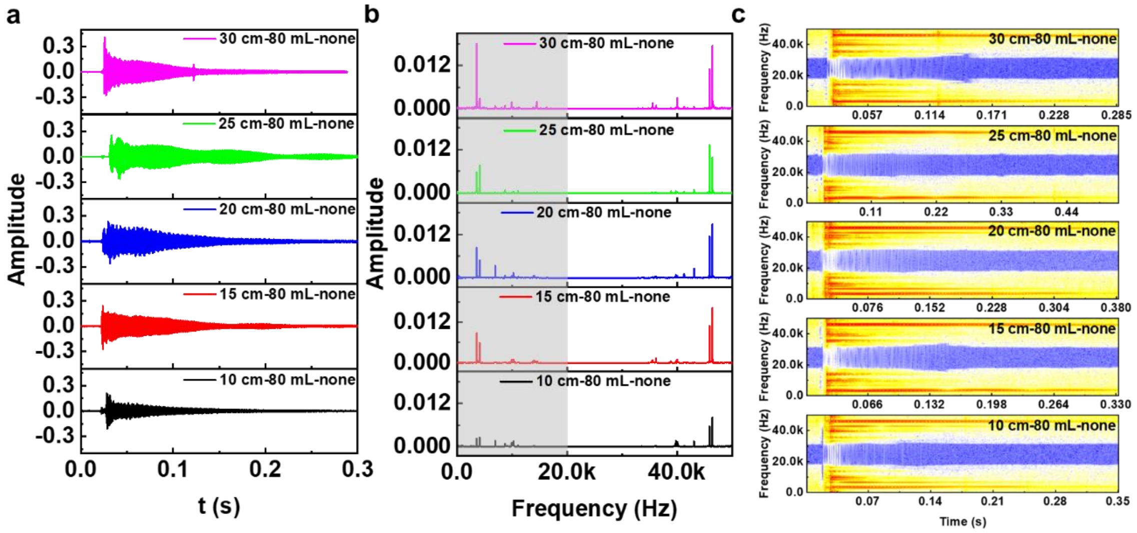

Fig. S12 The corresponding **a**, sound waves, **b**, fast Fourier transform (FFT) and **c**, short-time Fourier transform (STFT) of sounds in Supplementary Fig. S11

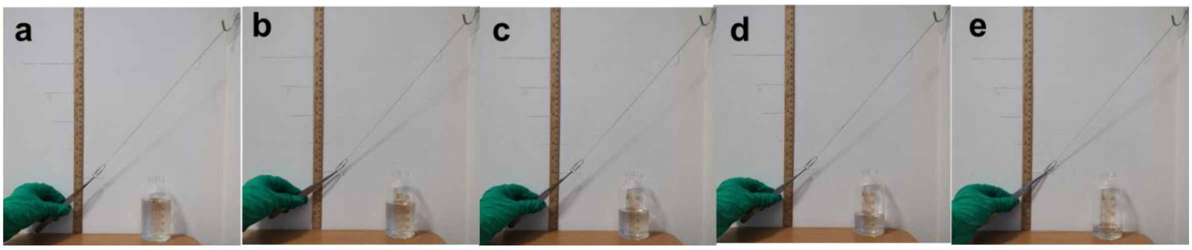

Fig. S13 The sounds with various tones. Water levels of **a**, 100 mL, **b**, 80 mL, **c**, 60 mL, **d**, 40 mL and **e**, 20 mL based on same knock height of 10 cm without bottle cap

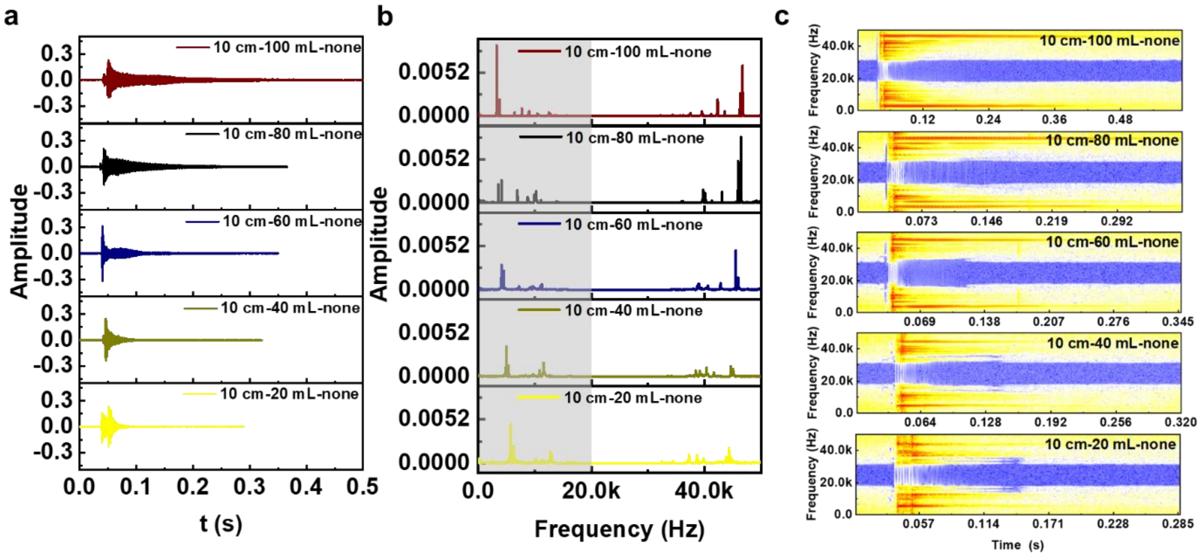

Fig. S14 The corresponding **a**, sound waves, **b**, FFT and **c**, STFT of sounds in Supplementary Fig. S13

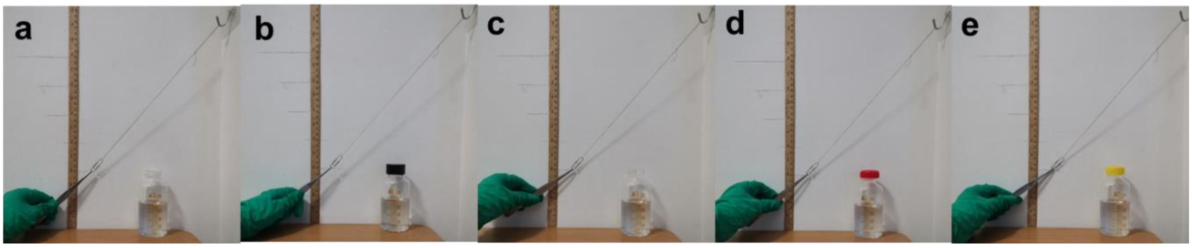

Fig. S15 The sounds with various timbre. Glass bottles with bottle cap of **a**, parafilm, **b**, dark, **c**, none, **d**, red and **e,** yellow based on same vertical height of 10 cm and 80 mL water

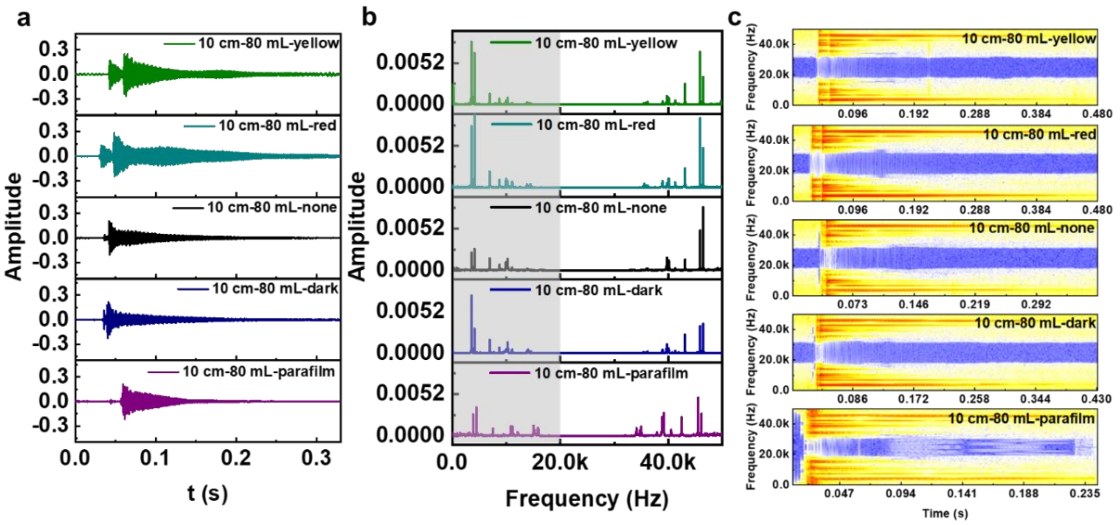

Fig. S16 The corresponding **a**, sound waves, **b**, FFT and **c**, STFT of sounds in Supplementary Fig. S15

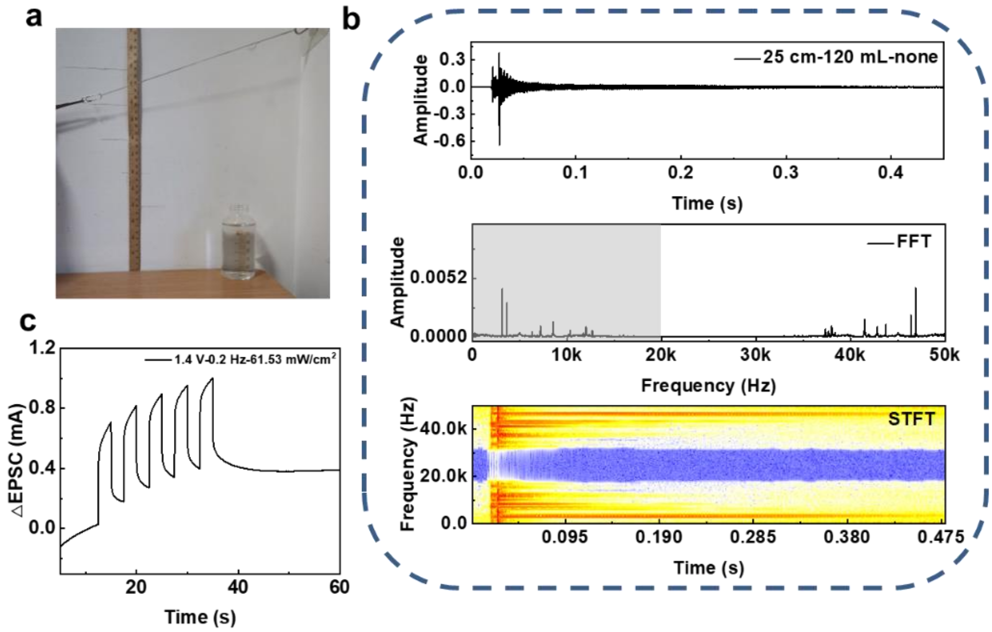

Fig. S17 A random sound at **a**, 25 cm of vertical height and 120 mL of water without the gap. **b**, The corresponding sound wave, FFT and STFT transform of **a**. **c**, The corresponding synaptic signal with input conditions at 1.4 V, 0.2 Hz and 61.53 mW/cm^2 of the **b**

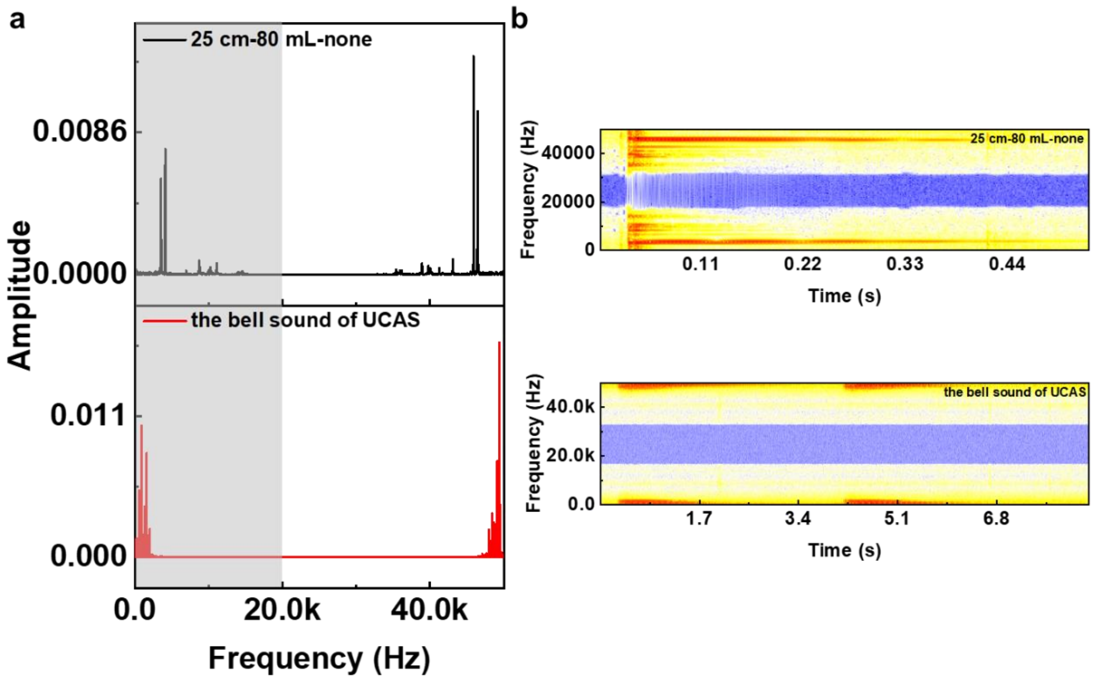

Fig. S18 **a**, Fast Fourier transforms (FFTs) and **b**, short-time Fourier transform (STFT) of sounds at 25 cm-80 mL-none and the bell sound of UCAS, respectively

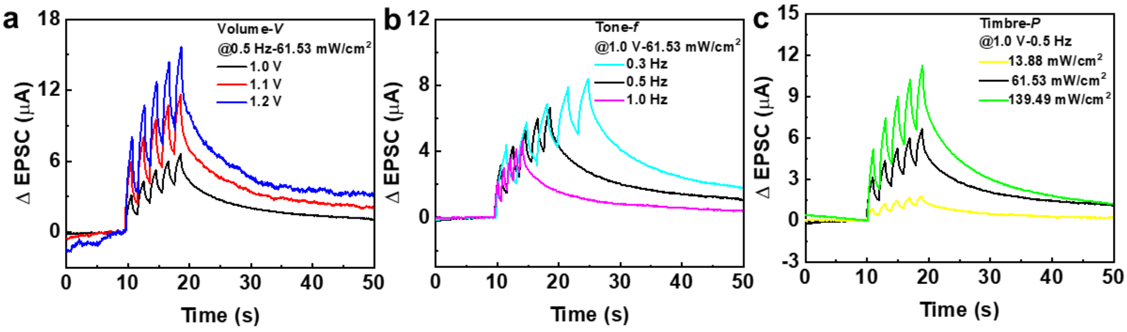

Fig. S19 The universality of **a**, volume-*V*, **b**, tone-*f*, **c,** timbre-*P* based on PBDB-T:ITIC system

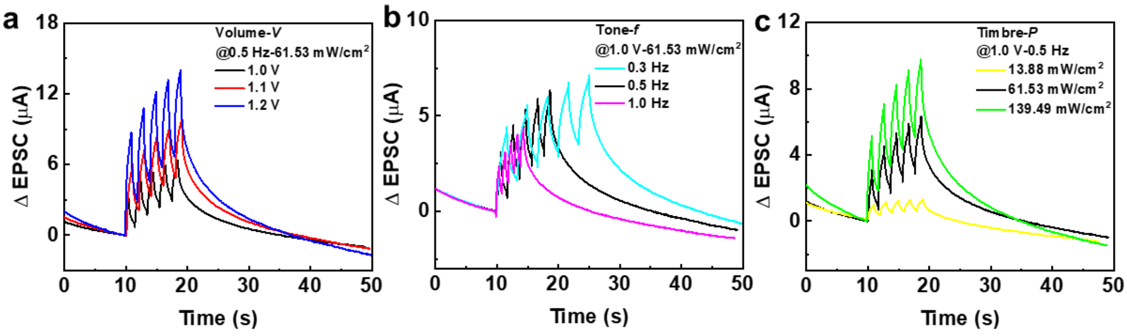

Fig. S20 The universality of **a**, volume-*V*, **b**, tone-*f*, **c**, timbre-*P* based on P3HT:$PC_{71}BM$ system

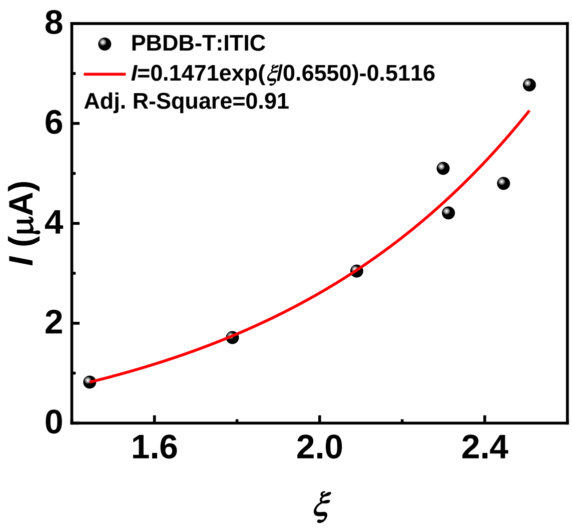

Fig. S21 Quantitative relationship of postsynaptic current (I) and recognition factor (ζ) for sound perception based on PBDB-T:ITIC system

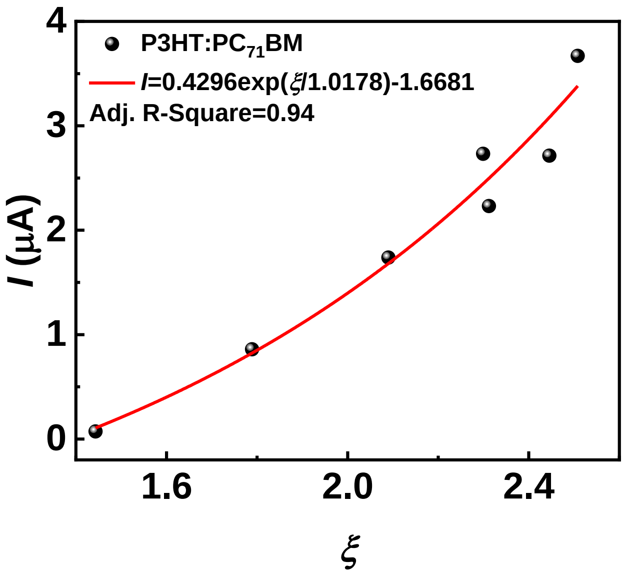

Fig. S22 Quantitative relationship of postsynaptic current (I) and recognition factor (ζ) for sound perception based on P3HT:$PC_{71}BM$ system

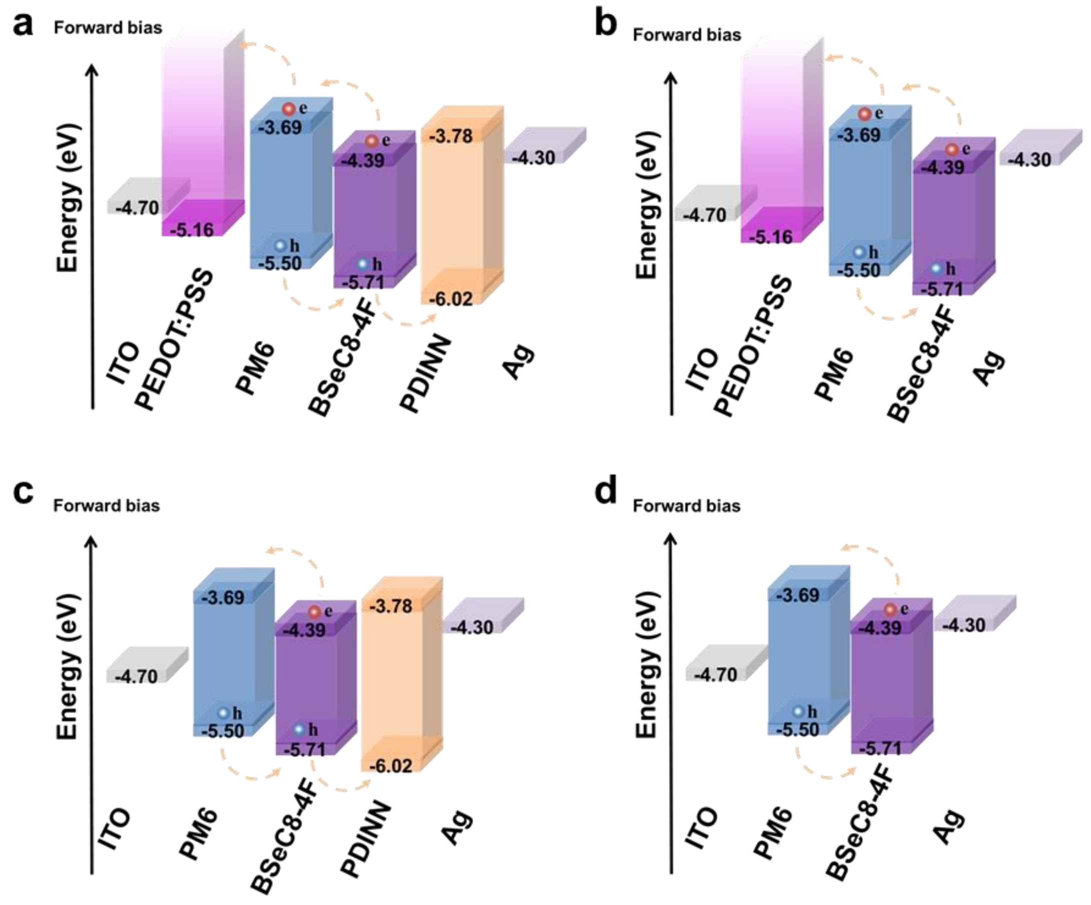

Fig. S23 Device structures of **a**, Device I (ITO/PEDOT:PSS/D:A/PDINN/Ag), **b**, Device II (ITO/PEDOT:PSS/D:A/Ag), **c**, Device III (ITO/D:A/PDINN/Ag) and **d**, Device IV (ITO/D:A/Ag)

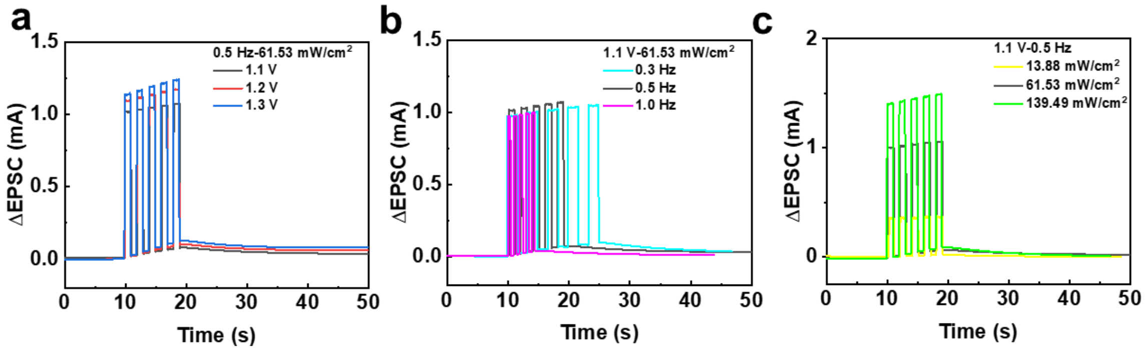

Fig. S24 The excitatory postsynaptic current (EPSC) of Device II with the change of **a**, voltage, **b**, frequency and **c**, light intensity at 850 nm

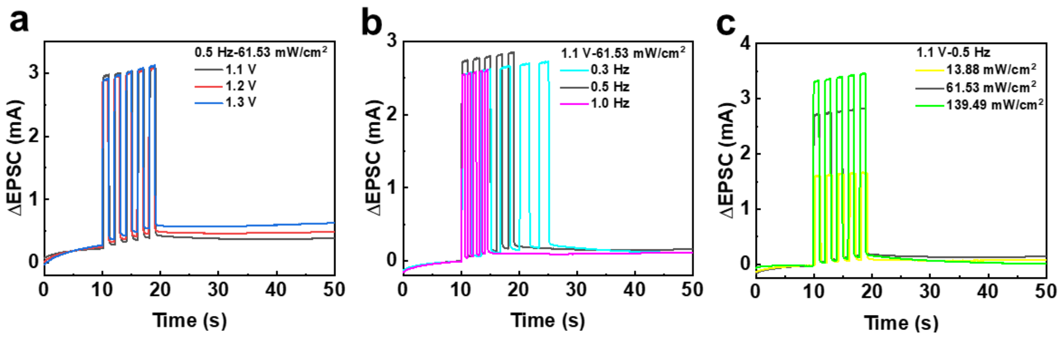

Fig. S25 The excitatory postsynaptic current (EPSC) of Device III with the change of **a**, voltage, **b**, frequency and **c**, light intensity at 850 nm

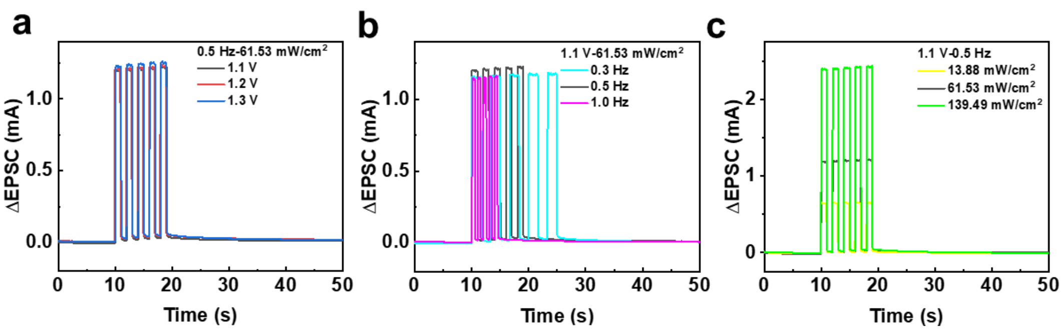

Fig. S26 The excitatory postsynaptic current (EPSC) of Device IV with the change of **a**, voltage, **b**, frequency and **c**, light intensity at 850 nm

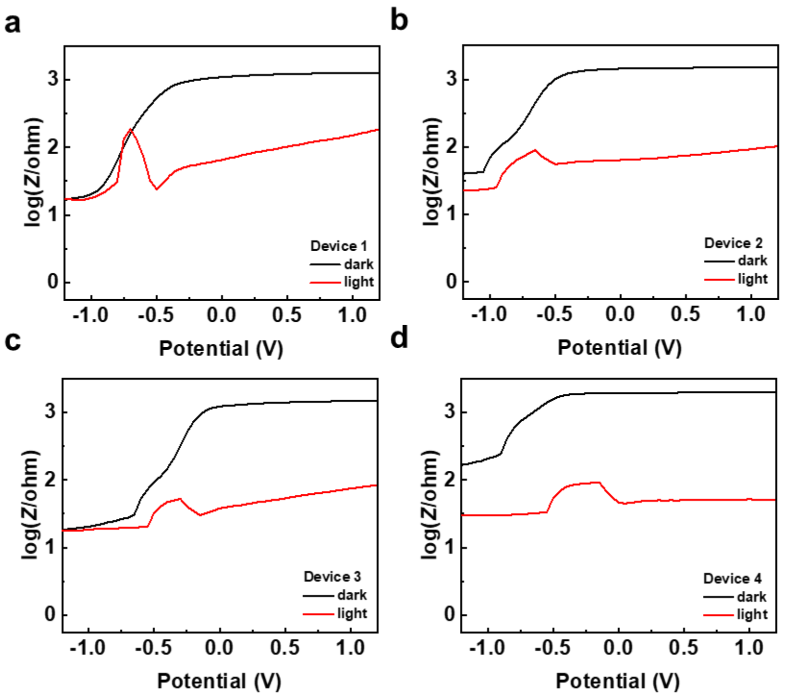

Fig. S27 The log*Z* value of Device I (ITO/PEDOT:PSS/D:A/PDINN/Ag) **a**, Device II (ITO/PEDOT:PSS/D:A/Ag) **b**, Device III (ITO/D:A/PDINN/Ag) **c**, and Device IV (ITO/D:A/Ag) **d**, under different bias voltages

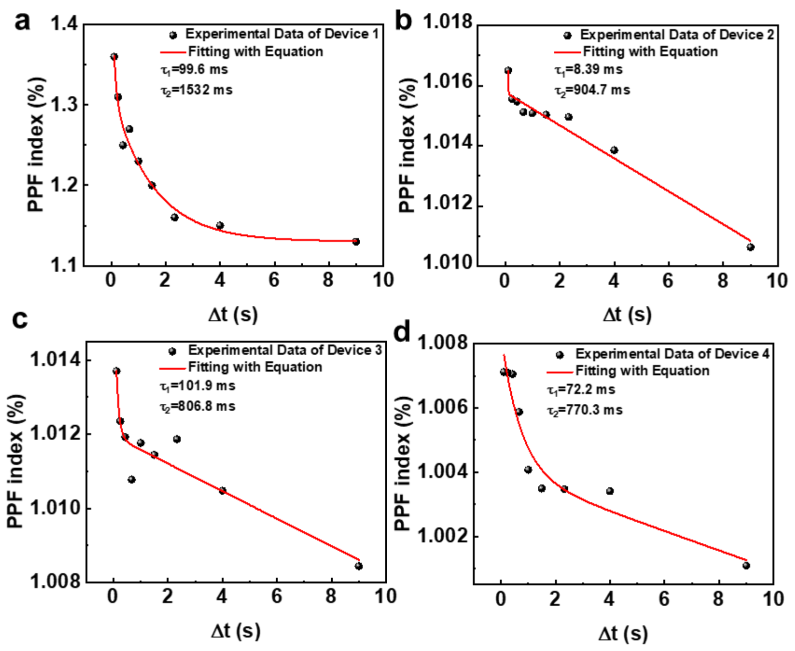

Fig. S28 PPF indexes of **a**, Device I, **b**, Device II, **c**, Device III and **d**, Device IV, respectively

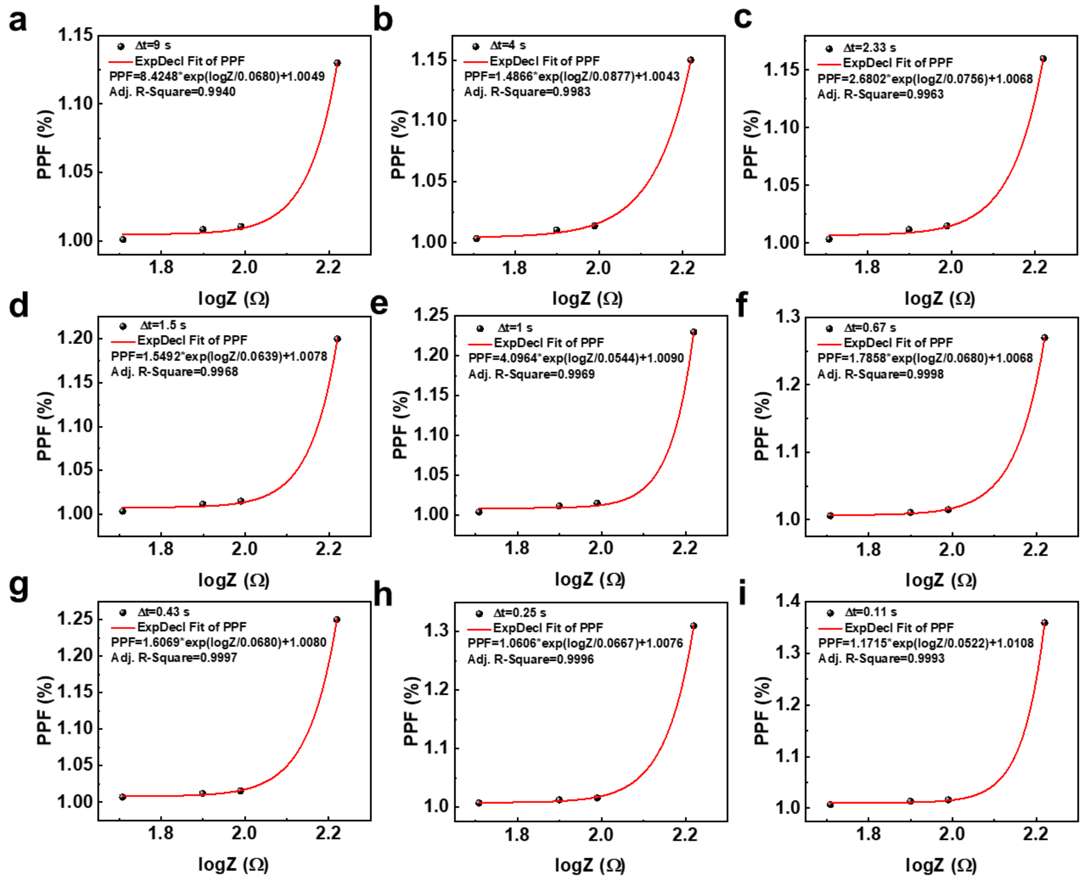

Fig. S29 Quantitative relationship curve of PPF index versus logZ plots at the light pulse pairs with interval of (Δt) **a** 9 s, **b** 4 s, **c** 2.33 s, **d** 1.5 s, **e** 1 s, **f** 0.67 s, **g** 0.43 s, **h** 0.25 s and **i** 0.11 s, respectively

S5 Supplementary Tables

Table S1 The modulation of sound signal via the input signal of voltage, frequency and light intensity of organic photoelectric synapse based on PBDB-T:ITIC and P3HT:$PC_{71}BM$ systems

Voltage (V)	Frequency (Hz)	Light intensity (mW/cm^2)	ζ	I (PBDB-T:ITIC)	I (P3HT:$PC_{71}BM$)	definition
Volume (amplitude)	Tone (frequency)	Timbre (waveform)				
1.0	0.5	61.53	2.0901	3.044	1.738	Sound a
1.1	0.5	61.53	2.2991	5.100	2.732	Sound b
1.2	0.5	61.53	2.5081	6.770	3.669	Sound c
1.0	0.3	61.53	2.3120	4.207	2.231	Sound d
1.0	1.0	61.53	1.7891	1.712	0.859	Sound e
1.0	0.5	13.88	1.4434	0.8201	0.072	Sound f
1.0	0.5	139.49	2.4456	4.798	2.714	Sound g

Table S2 The values of logZ under different bias voltages at light stimuli (850 nm, 61.53 mW/cm^2)

Devices	-1.1 V	-1.0 V	0 V	0.8 V	1.0 V	1.1 V
Device 1	1.2240	1.2509	1.8140	2.1089	2.1767	2.2196
Device 2	1.3619	1.3811	1.8068	1.9268	1.9670	1.9898
Device 3	1.2553	1.2676	1.5800	1.8174	1.8742	1.8993
Device 4	1.4822	1.4793	1.6628	1.7036	1.7137	1.7082

S6 ^{1}H and ^{13}C NMR Spectra of the Key Intermediates and Final Products

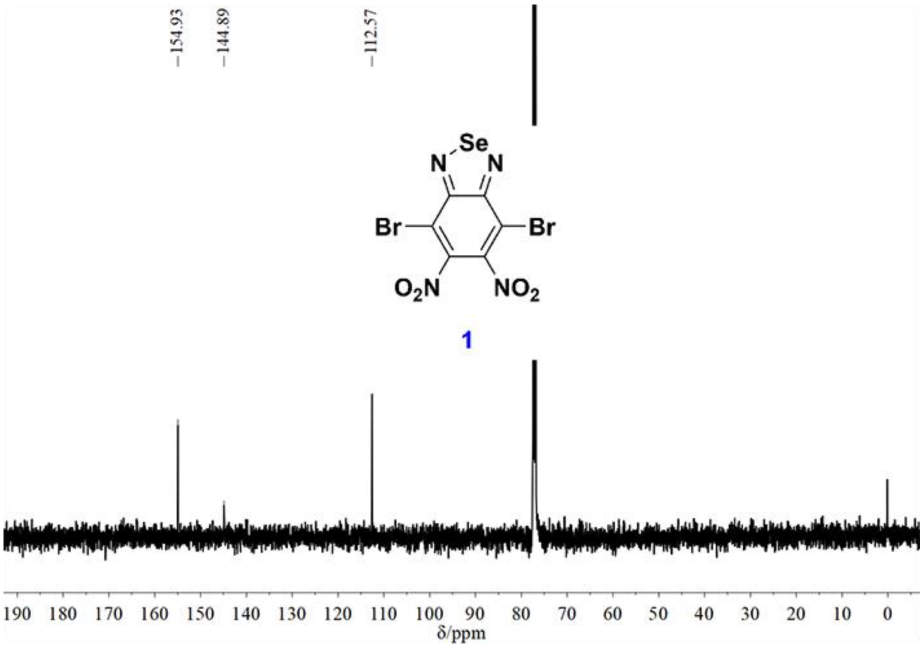

Fig. S30 ^{13}C NMR (126 MHz) of compound **1**

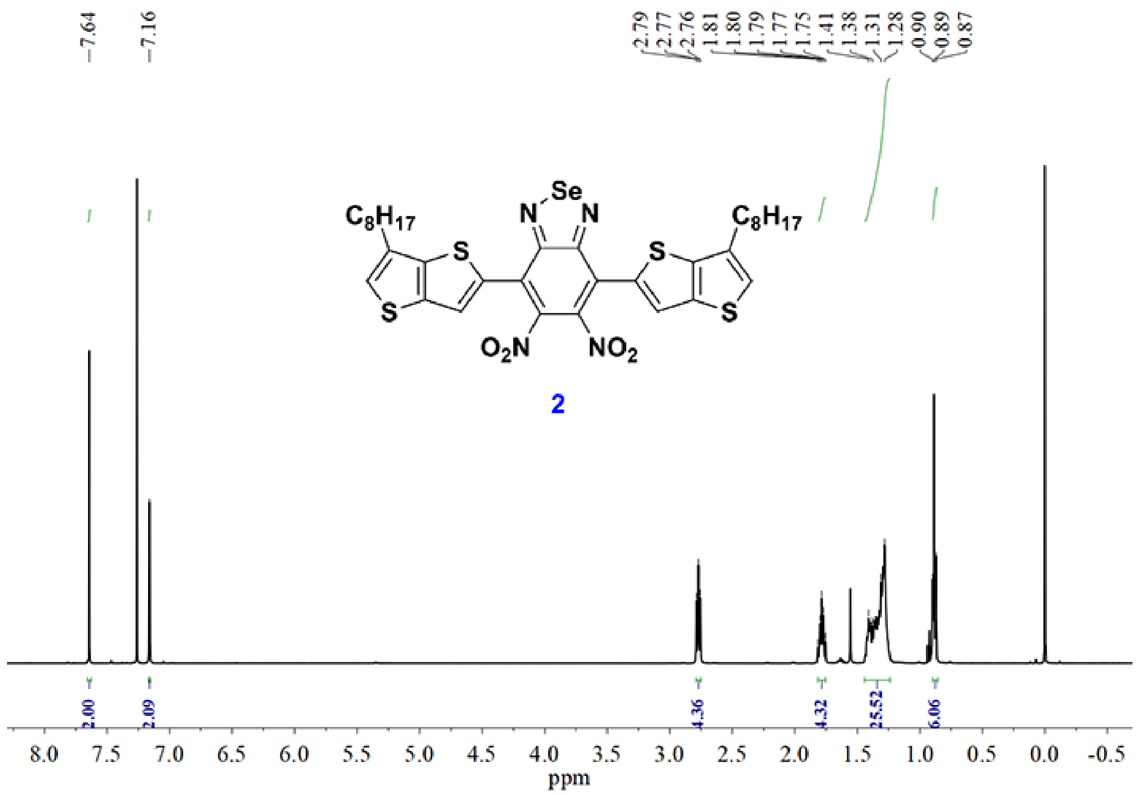

Fig. S31 ^{1}H NMR (500 MHz) of compound **2**

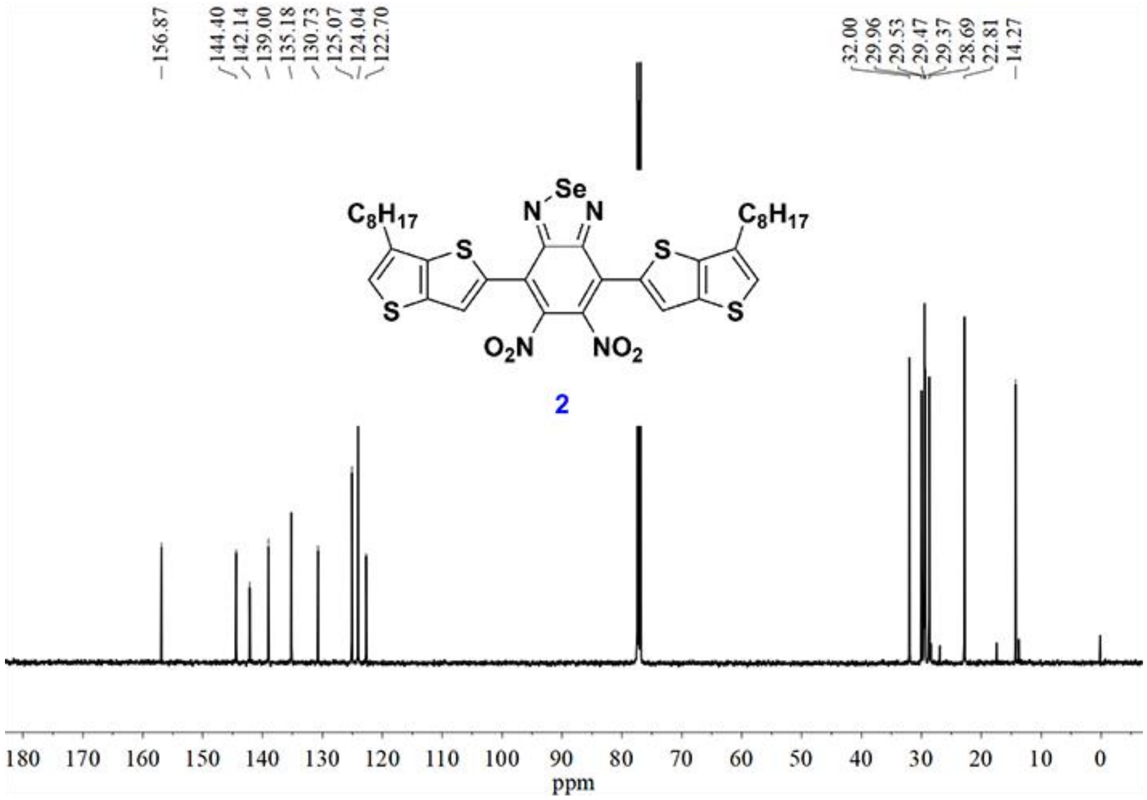

Fig. S32 ^{13}C NMR (126 MHz) of compound **2**

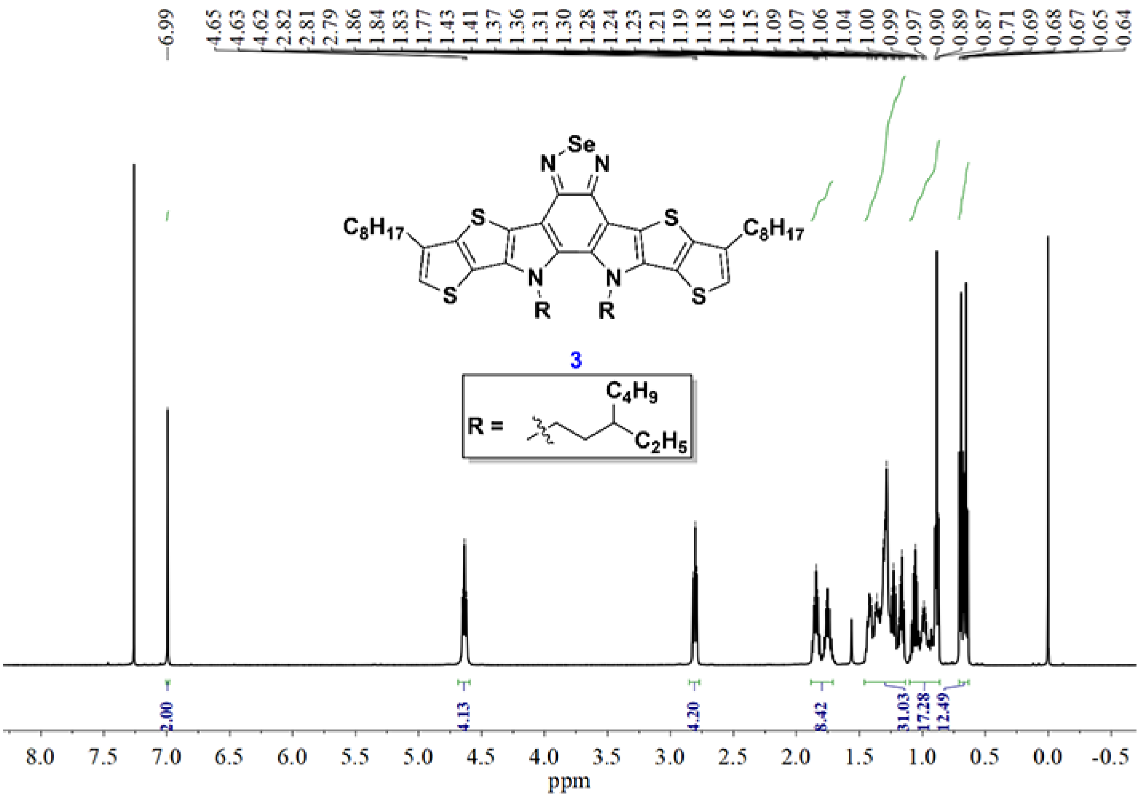

Fig. S33 ^{1}H NMR (500 MHz) of compound **3**

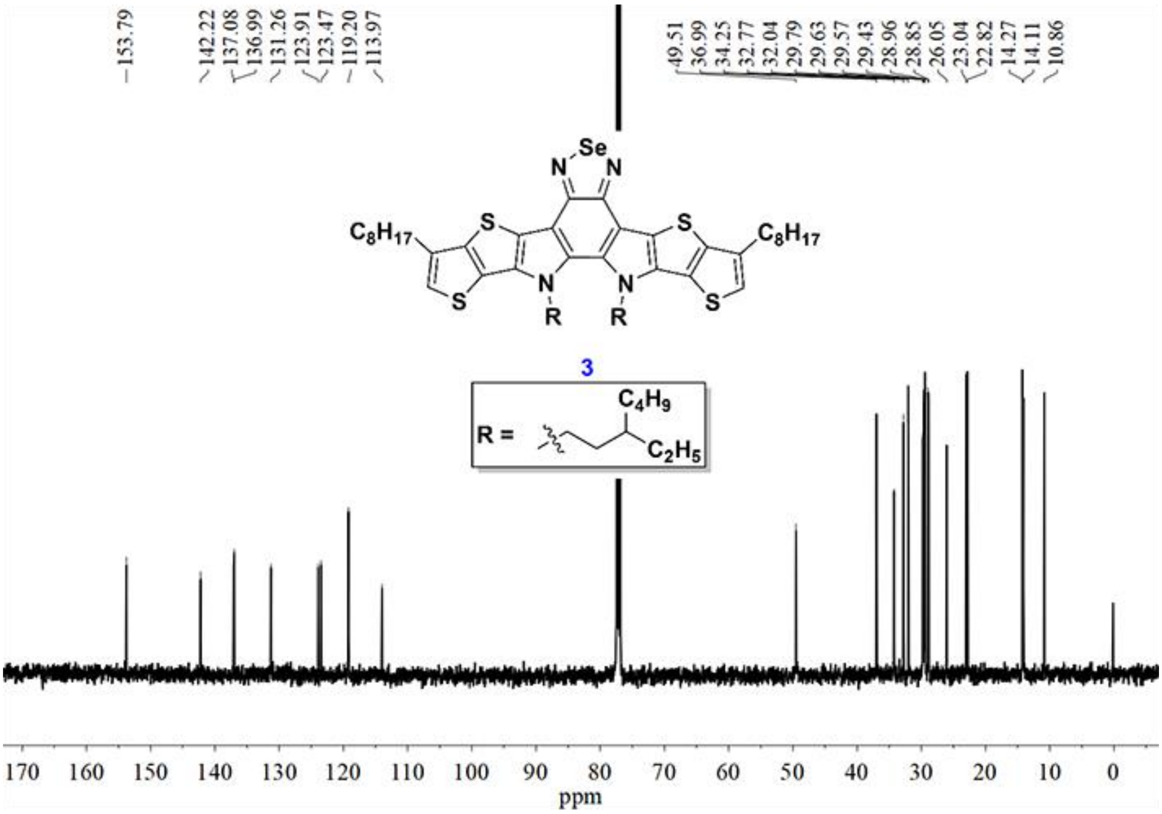

Fig. S34 ^{13}C NMR (126 MHz) of compound **3**

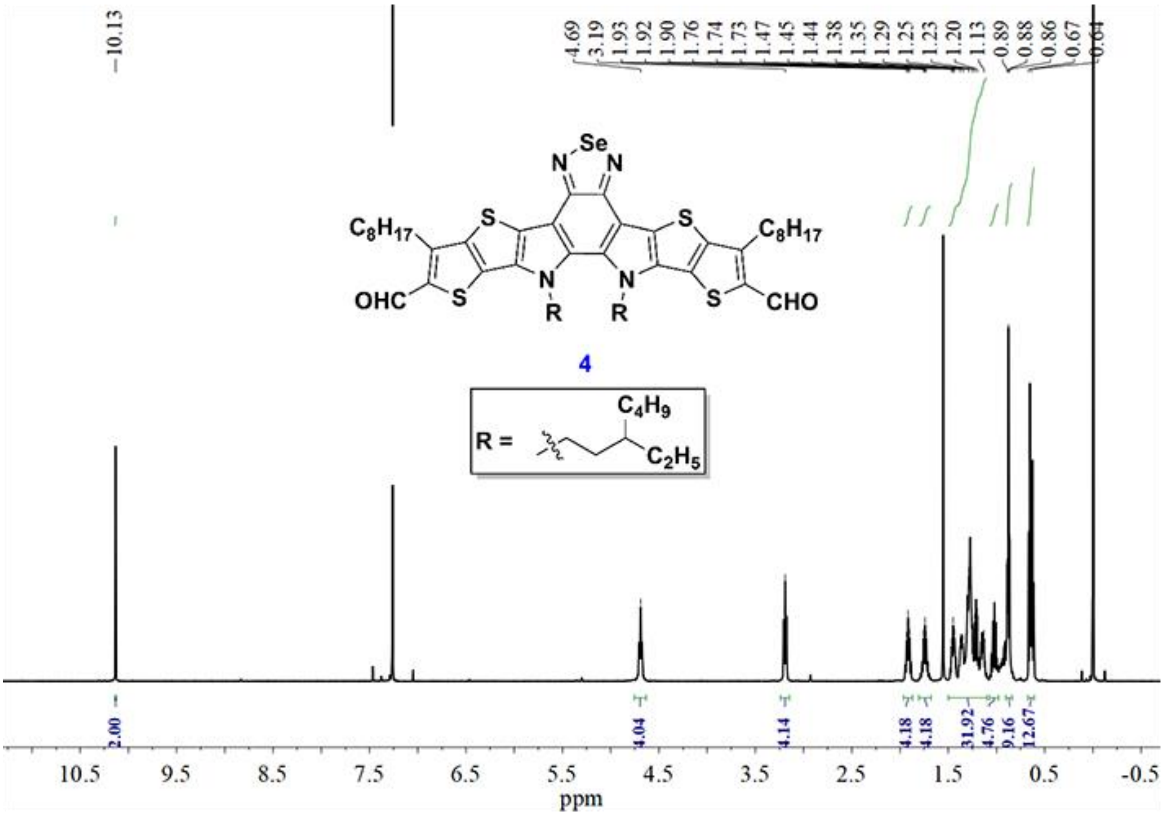

Fig. S35 ^{1}H NMR (500 MHz) of compound **4**

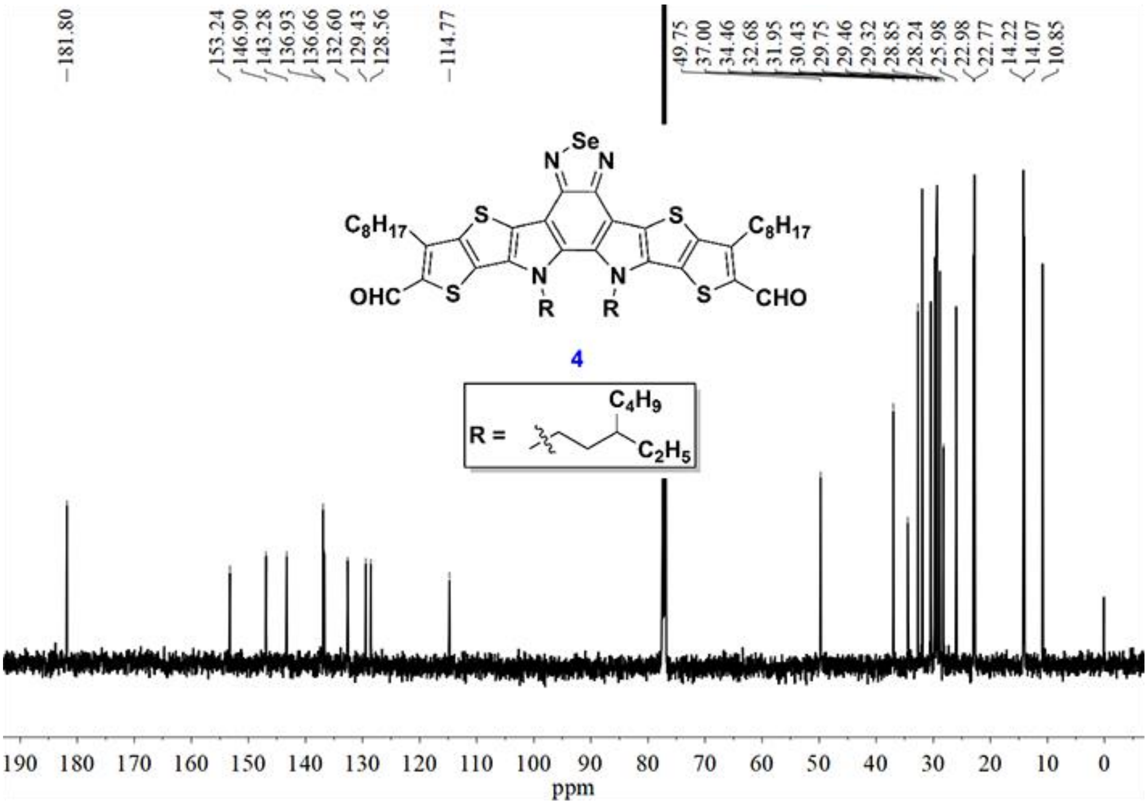

Fig. S36 ^{13}C NMR (126 MHz) of compound **4**

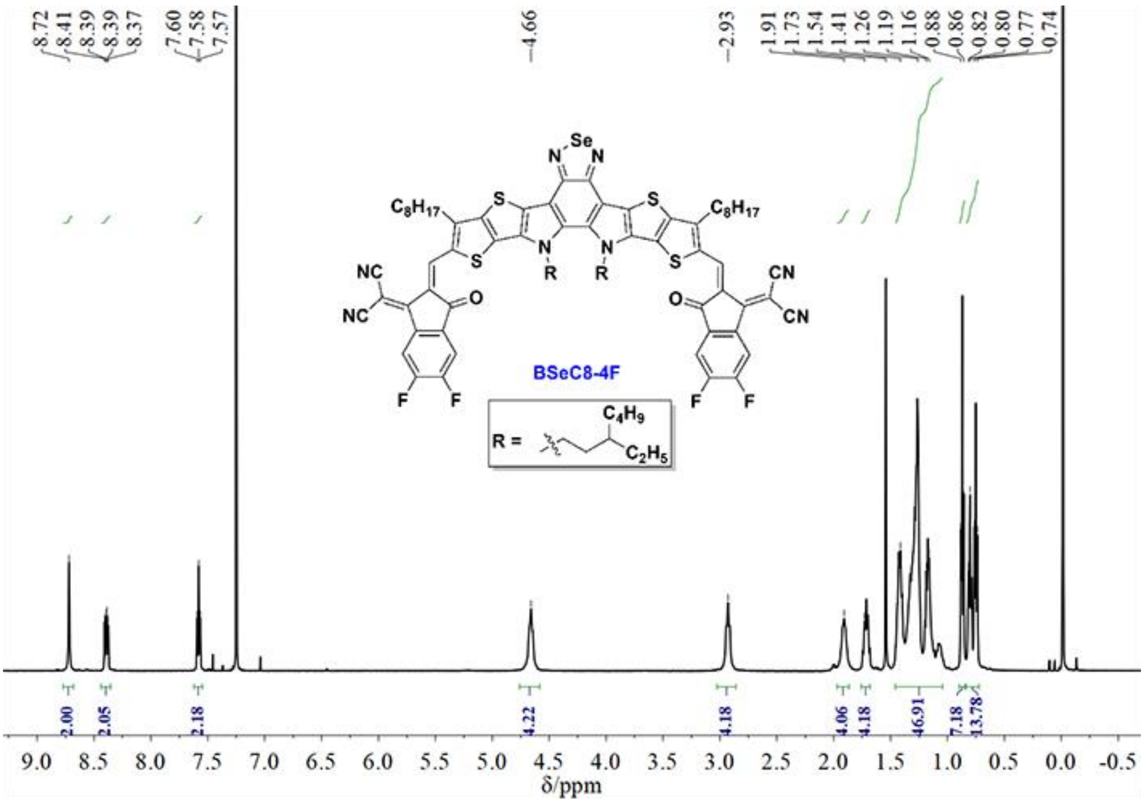

Fig. S37 ^{1}H NMR (500 MHz) of **BSeC8-4F**

Supplementary References

[S1] W.-Q. Zhang et al., Robust metal–organic framework containing benzoselenadiazole for highly efficient aerobic cross-dehydrogenative coupling reactions under visible light. Inorg. Chem. **55**, 1005-1007 (2016). https://doi.org/10.1021/acs.inorgchem.5b02626
